# Breaking down barriers: comprehensive functional analysis of the *Aspergillus niger* chitin synthase repertoire

**DOI:** 10.1186/s40694-024-00172-7

**Published:** 2024-03-11

**Authors:** Lars Barthel, Timothy Cairns, Sven Duda, Henri Müller, Birgit Dobbert, Sascha Jung, Heiko Briesen, Vera Meyer

**Affiliations:** 1 Chair of Applied and Molecular Microbiology, Institute of Biotechnology, Technische Universität Berlin, Berlin, Germany; 2 School of Life Sciences Weihenstephan, Chair of Process Systems Engineering, Technical University of Munich, Freising, Germany

**Keywords:** *Aspergillus niger*, Chitin, Cell wall, µCT, Protein secretion, Macromorphology, Pellet, Biotechnology

## Abstract

**Background:**

Members of the fungal kingdom are heterotrophic eukaryotes encased in a chitin containing cell wall. This polymer is vital for cell wall stiffness and, ultimately, cell shape. Most fungal genomes contain numerous putative chitin synthase encoding genes. However, systematic functional analysis of the full chitin synthase catalogue in a given species is rare. This greatly limits fundamental understanding and potential applications of manipulating chitin synthesis across the fungal kingdom.

**Results:**

In this study, we conducted *in silico* profiling and subsequently deleted all predicted chitin synthase encoding genes in the multipurpose cell factory *Aspergillus niger*. Phylogenetic analysis suggested nine chitin synthases evolved as three distinct groups. Transcript profiling and co-expression network construction revealed remarkably independent expression, strongly supporting specific role(s) for the respective chitin synthases. Deletion mutants confirmed all genes were dispensable for germination, yet impacted colony spore titres, chitin content at hyphal septa, and internal architecture of submerged fungal pellets. We were also able to assign specific roles to individual chitin synthases, including those impacting colony radial growth rates (ChsE, ChsF), lateral cell wall chitin content (CsmA), chemical genetic interactions with a secreted antifungal protein (CsmA, CsmB, ChsE, ChsF), resistance to therapeutics (ChsE), and those that modulated pellet diameter in liquid culture (ChsA, ChsB). From an applied perspective, we show *chsF* deletion increases total protein in culture supernatant over threefold compared to the control strain, indicating engineering filamentous fungal chitin content is a high priority yet underexplored strategy for strain optimization.

**Conclusion:**

This study has conducted extensive analysis for the full chitin synthase encoding gene repertoire of *A. niger*. For the first time we reveal both redundant and non-redundant functional roles of chitin synthases in this fungus. Our data shed light on the complex, multifaceted, and dynamic role of chitin in fungal growth, morphology, survival, and secretion, thus improving fundamental understanding and opening new avenues for biotechnological applications in fungi.

**Supplementary Information:**

The online version contains supplementary material available at 10.1186/s40694-024-00172-7.

## Background

Across the many life modes, morphologies, and environments in which fungi proliferate and colonize, chitin is a crucial molecule that provides rigidity and stiffness to the cell wall [1]. This structure, in turn, ultimately defines cell shape while offering physical protection from the external environment [1–3]. Chitin consists of N-acetylglucosamine units linked by β-(1 → 4) glycosidic bonds which form long-chain polymers that assemble into microfibrils. Although the diversity in structure and composition of the cell wall is substantial throughout the fungal kingdom (reviewed in [2]), it is broadly true that a complex mesh of chitin and 1,3-β-D-glucan chains are linked to form a rigid inner layer. The outer layer, in contrast, may consist of 1,6-β-glucan, 1,4-β-glucan, α-1,3-glucan, mannan, galactomannan, galactosamine, glycosaminoglycans, melanins, and various proteins [1–3]. The cell wall is dynamic and multifaceted: robust yet flexible, porous yet impermeable, adhesive yet repellent, with the ability to sense the environment and, additionally, reinforce itself by the activation of the cell wall integrity pathway. Much of this cell wall reinforcement occurs by rapid production of various structural molecules, including chitin [4].

The enzymes that biosynthesize chitin precursors are largely conserved across the fungal kingdom [5], with key steps of the pathway being glucose → fructose-6-phosphate → glucosamine-6-phosphate → uridine diphosphate N-acetylglucosamine (UDP-GlcNAc). The final step of chitin synthesis requires chitin synthases, which add UDP-GlcNAc monomers to a growing chitin chain [2]. These enzymes are anchored to the plasma membrane at specific subcellular locations (e.g., hyphal tip, septum, bud site, etc. [6–8]). Transport to the plasma membrane can occur via the classical secretion route, where chitin synthases are packaged into vesicles alongside other cell wall synthesizing enzymes [6, 8]. Some chitin synthase genes encode a myosin motor domain (MMD) at the N-termini. Genetic targeting of the MMD causes mis-localization, although it is hypothesised that this does not directly transport the synthase to the action site, but rather supports exocytosis by attaching secretory vesicles to actin fibres (reviewed in [9]).

Interestingly, the number of predicted chitin synthase encoding genes varies drastically between different species, ranging from a single gene in fission yeast to over thirty in some sequenced fungi [10]. Evolutionary expansion of chitin synthase encoding genes occurred alongside the emergence of the filamentous life mode, thus implicating their role in the adaptation of fungal multicellularity and hyphal formation [11].

Given the fundamental role of chitin in cell wall stiffness and rigidity across the fungal kingdom, interest in this molecule and its biosynthetic enzymes/encoding genes span medical mycology, plant pathology, and industrial biotechnology [1–3]. Chemical inhibition of chitin synthases has offered therapeutic promise in the clinic, e.g., by nikkomycins [12]. During plant infection, degradation of pathogen chitin by host chitinases and subsequent detection of chitin fragments by resistance proteins represents a potential strategy to engineer crop immunity to fungal infection [13]. For industrial biotechnology, several avenues of research have genetically targeted chitin synthesis- most commonly to generate strains with optimized submerged morphologies [14].

For example, RNAi knockdown of a predicted chitin synthase in the antibiotic producer *Penicillium chrysogenum* drastically impacted strain morphology [15]. Notably, mutants displayed a hyperbranched hyphal phenotype on solid agar, and in liquid culture produced large pellets with elevated titres of penicillin [15]. This study is consistent with the growing body of evidence that manipulating genes encoding cell wall biosynthetic enzymes, including chitin synthases, can modify submerged morphologies and enhance product titres during fermentation.

Another possibility that makes disruption of chitin synthase encoding genes especially attractive for industrial applications is that a desired product may more readily pass into the reaction media in isolates with defective cell wall. Indeed, we have demonstrated that enhanced sensitivity to chitin-based cell wall perturbation (Congo red) was correlated with increased total protein titres in the multipurpose cell factory *Aspergillus niger* [16]. In this study, six genes with a predicted role in morphology (but, notably, no direct role in chitin synthesis) were placed under control of the tetracycline conditional expression system Tet-on [16]. By phenotypically screening mutants on solid and liquid culture, and building these data into regression models, we were able to predict that total protein titres during shake flask fermentation were enhanced by robust growth, high resistance to temperature stress, small pellets, and, importantly, sensitivity to chitin perturbation [16]. Finally, given that chitin synthases can be delivered to the plasma membrane in secretory vesicles, knock-out strains may have reduced vesicle cargo load. Thus, increased protein flux through the classical pathway may occur in chitin synthase null mutants. Taken together, we reasoned that chitin synthase encoding genes are high priority yet underexplored targets for strain engineering in *A. niger*.

The aim of this study was to comprehensively profile the chitin synthase repertoire of *A. niger*. We conducted a number of *in silico* analyses, including constructing phylogenetic trees and co-expression networks, both of which strongly suggest that nine predicted chitin synthase encoding genes perform a range of redundant or non-redundant functions in this fungus. We generate a suite of respective chitin synthase knock out strains, and conduct quantitative phenotypic screens on a range of solid and liquid media. These data demonstrate various defects in cell growth, colony development, and changes to cell wall composition. For the first time, we utilize a recently developed µCT imaging protocol to profile internal pellet core architecture from mutant isolates, revealing chitin synthase gene deletion has multiple impacts on submerged macromorphology. Finally, deletion of *chsF* resulted in elevated titres of total extracellular protein relative to the control isolate in shake flask models of fermentation, strongly suggesting that chitin synthases are a promising yet underexplored avenue for maximizing efficiency of fungal cell factories.

## Results

### Phylogenetic analysis suggests three major groups of *A. niger* chitin synthase encoding genes

**Fig. 1:**
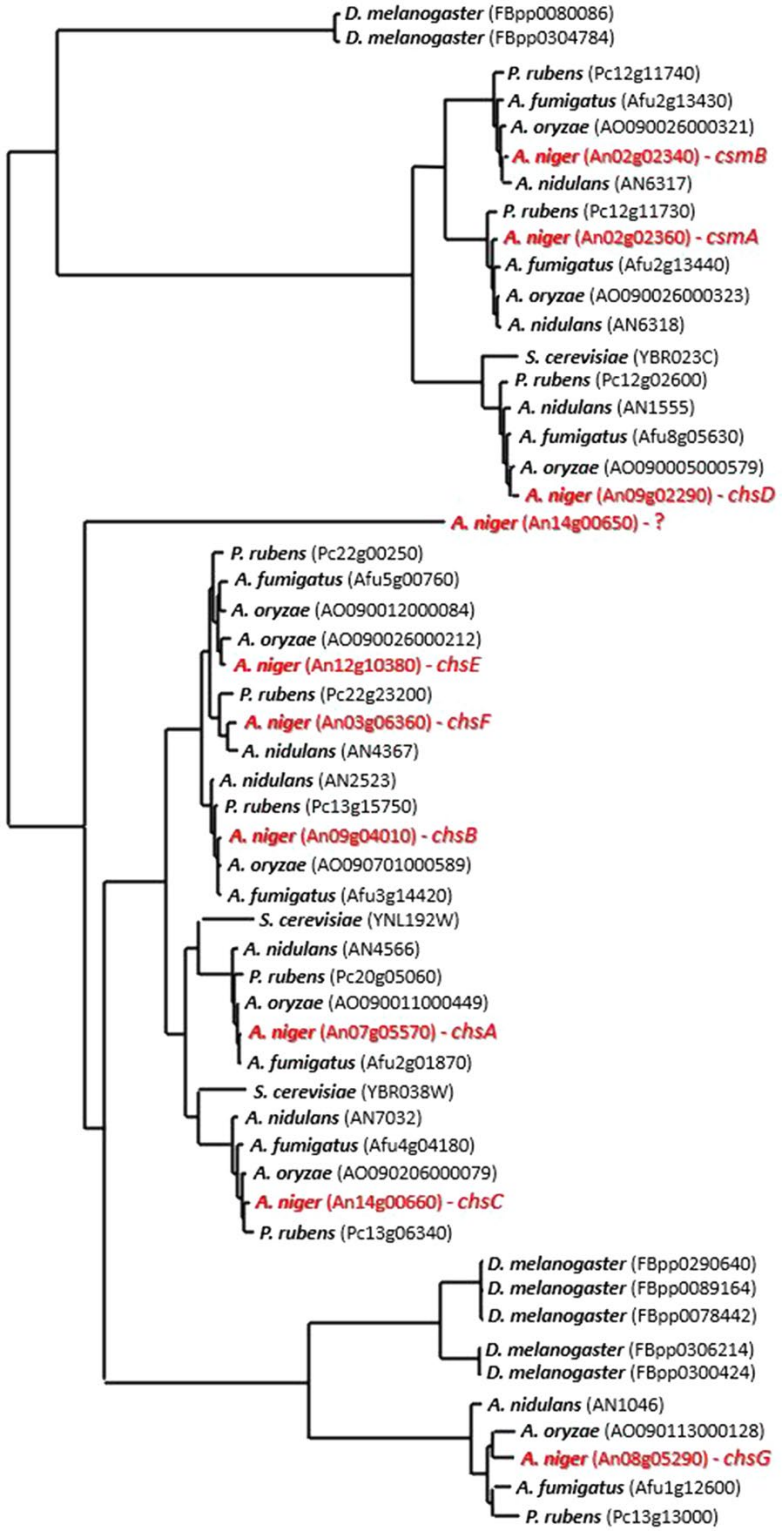
Phylogenetic tree of predicted chitin synthase protein sequences. Species name and corresponding ORF code are given. *A. niger* genes are highlighted in red. Note ORF An14g00650 was identified in this analysis and subsequently discredited as a chitin synthase encoding gene (see main text)

In order to catalogue the full predicted chitin synthase encoding gene repertoire of *A. niger*, we searched for chitin synthase domains in the CBS 513.88 genome [17]. All ten returned hits were analysed in a phylogenetic analysis that suggested three groups: (i) *chsD*, *csmA*, *csmB* (ii) *chsA*, *chsB*, *chsC*, *chsE*, *chsF* and (iii) *chsG* (Fig. 1). Note, gene An14g00650 was discontinued from further analysis as it contained only 124 amino acids and lacked conserved chitin synthase domains following more extensive *in silico* analysis (data not shown). We hypothesized that groups (i) and (ii) (Fig. 1) might play related biological roles, an observation that further reinforced the need to functionally analyse all chitin synthase encoding genes in *A. niger*. Interestingly, groups (i) and (ii) included predicted chitin synthases from *Saccharomyces cerevisiae* and *Penicillium rubens*, whereas the *chsG* group only contained genes from *Penicillium rubens*, other Aspergilli, and *Drosophila melanogaster* (Fig. 1). Orthology searches confirmed *chsG* was absent in common environmental yeasts, including *Schizosaccharomyces pombe*, *Pichia pastoris*, and *Candida auris.* Grouping of *chsG* with chitin synthase encoding genes from filamentous fungi and *D. melanogaster*, yet not yeast, may suggest a functional role for *chsG* that is conserved for hyphal forming fungi and insects. An alternative explanation is evolution from a progenitor chitin synthase that was important for insects but broadly dispensable for members of the fungal kingdom, hence loss of this gene in *S. cerevisiae*.

The resulting nine putative synthases (Fig. 1) are concordant with annotation from whole genome sequencing [17]. Chitin synthase, glycosyltransferase, and motor domains were analysed across these genes (Additional file 1: Figure S1 and Additional file 1: Table S1). *chsA-F* contained chitin synthase and/or glycosyltransferase domains, whereas *csmA* and *csmB* additionally encode a motor domain (Additional file 1: Figure S1). Most genes are physically distributed across *A. niger* chromosomes, whereas *csmA* and *csmB* are located in close proximity (< 4 kb apart), suggesting a gene duplication event. As expected, amino acid sequence BLASTs of the nine genes reveals high concordance with industrial (*Aspergillus oryzae*), model (*Aspergillus nidulans*) and human infecting (*Aspergillus fumigatus*) Aspergilli, with the notable exception of *chsF* (*A. fumigatus, A. oryzae*) and *chsE* (*A. nidulans*, Additional file 1: Table S2). Therefore, the *A. niger* chitin synthase encoding gene repertoire is notably higher than model yeast systems [10], and in general well conserved in this genus.

### *A. niger* chitin synthase encoding genes are embedded in unique transcriptional networks

We have previously conducted a meta-analysis of *A. niger* transcription using microarrays across 155 unique cultivation conditions [18]. Seven chitin synthases were robustly expressed in this dataset, whereas *chsG* and *chsF* were rarely transcriptionally active and expressed at low levels (Additional file 1: Fig. S2). From these data, we generated co-expression networks for each *A. niger* chitin synthase encoding gene as described previously [19–21], whereby any gene pair with comparable transcriptional signatures across the microarray meta-analysis (Spearman correlation coefficient > 0.5) were considered coexpressed. This approach has previously enabled accurate predictions of gene function based on the co-expression network in which a gene of interest is embedded [19–21]. *chsF* was the only candidate that was not co-expressed with any *A. niger* gene, which is due to the very low and rare expression of this ORF. By probing the eight generated co-expression networks for enriched GO terms, we were able to assign various functional or subcellular predictions for each gene, with the exception of *chsB* network which was not significantly enriched in any GO term (Additional file 1: Figures S3 and S4). These predictions included but were not limited to *csmA* (site of polarized growth), *csmB* (spore bearing structure development), *chsA* (cell wall organization), *chsC* (mitotic cell cycle progress), *chsD* (cell septum), *chsE* (endomembrane system), and *chsG* (DNA recombination). In general, the networks and respective enriched GO terms were distinct for each chitin synthase encoding gene, strongly suggesting their transcriptional deployment of chitin synthase genes in *A. niger* is largely independent. Indeed, only two from thirty-six pair wise comparisons were above our threshold for co-expression (*chsD* and *csmB*, *chsB* and *chsD*, Spearman > 0.5, Additional file 1: Figure S5). Taken together, transcriptional analysis supports the notion that chitin synthases are not functionally redundant in *A. niger* and may play specific roles in this fungus.

### All *A. niger* chitin synthase mutants can germinate yet are defective in colony growth or development

In order to functionally analyse the nine putative *A. niger* chitin synthase encoding genes, we began by constructing individual deletion mutants in progenitor isolate MA169.4 [22] using a split marker approach (Table 1). All genes were successfully deleted using this method, with the exception of *chsC*, which was recalcitrant to targeting despite numerous transformation attempts. Thus, we applied genome editing to successfully generate a Δ*chsC* strain (see Methods). A Δ*chsE* Δ*chsD* double mutant was constructed at a later stage in this study after analysis of cell wall composition of the respective single mutants. For clarity, we report phenotypic screening of this strain alongside the nine single mutants. Replacement of native ORFs with the transformation marker was confirmed by PCR. Southern blot analysis was used as a second quality control step to confirm targeting of the exogenous DNA at the desired locus and the absence of ectopic integration (Additional file 1: Figure S6). Strains generated in this study are listed in Table 1.

**Table 1:** *A. niger* strains used in this study

Name	Genotype	Notes	Background isolates	Reference
MA169.4	*kusA*::DR*-amdS-*DR*, pyrG *^*−*^	Progenitor strain for mutant construction	N402, AB4.1	Carvalho et al. [22]
MJK17.25	*kusA*::DR*-amdS-*DR*, pyrG* +	*pyrG* positive control strain	N402, AB4.1, MA169.4	Schäpe et al. [18]
LB7.1	*kusA*::DR*-amdS-*DR*, pyrG* + , Δ*csmB*	–	N402, AB4.1, MA169.4	This study
LB8.18	*kusA*::DR*-amdS-*DR*, pyrG* + , Δ*csmA*	–	N402, AB4.1, MA169.4	This study
LB9.1	*kusA*::DR*-amdS-*DR*, pyrG* + , Δ*chsF*	–	N402, AB4.1, MA169.4	This study
LB10.1	*kusA*::DR*-amdS-*DR*, pyrG* + , Δ*chsA*	–	N402, AB4.1, MA169.4	This study
LB11.1	*kusA*::DR*-amdS-*DR*, pyrG* + , Δ*chsG*	–	N402, AB4.1, MA169.4	This study
LB12.14	*kusA*::DR*-amdS-*DR*, pyrG* + , Δ*chsD*	–	N402, AB4.1, MA169.4	This study
LB13.3	*kusA*::DR*-amdS-*DR*, pyrG* + , Δ*chsB*	–	N402, AB4.1, MA169.4	This study
LB14.1	*kusA*::DR*-amdS-*DR*, pyrG* + , Δ*chsE*	–	N402, AB4.1, MA169.4	This study
SD1.3	*kusA*::DR*-amdS-*DR*, pyrG* + , Δ*chsC*	–	N402, AB4.1, MA169.4	This study
ME1.4	*kusA*::DR*-amdS-*DR*, pyrG* + , Δ*chsE*, Δ*chsD*	–	N402, AB4.1, MA169.4, LB14.1	This study

**Fig. 2:**
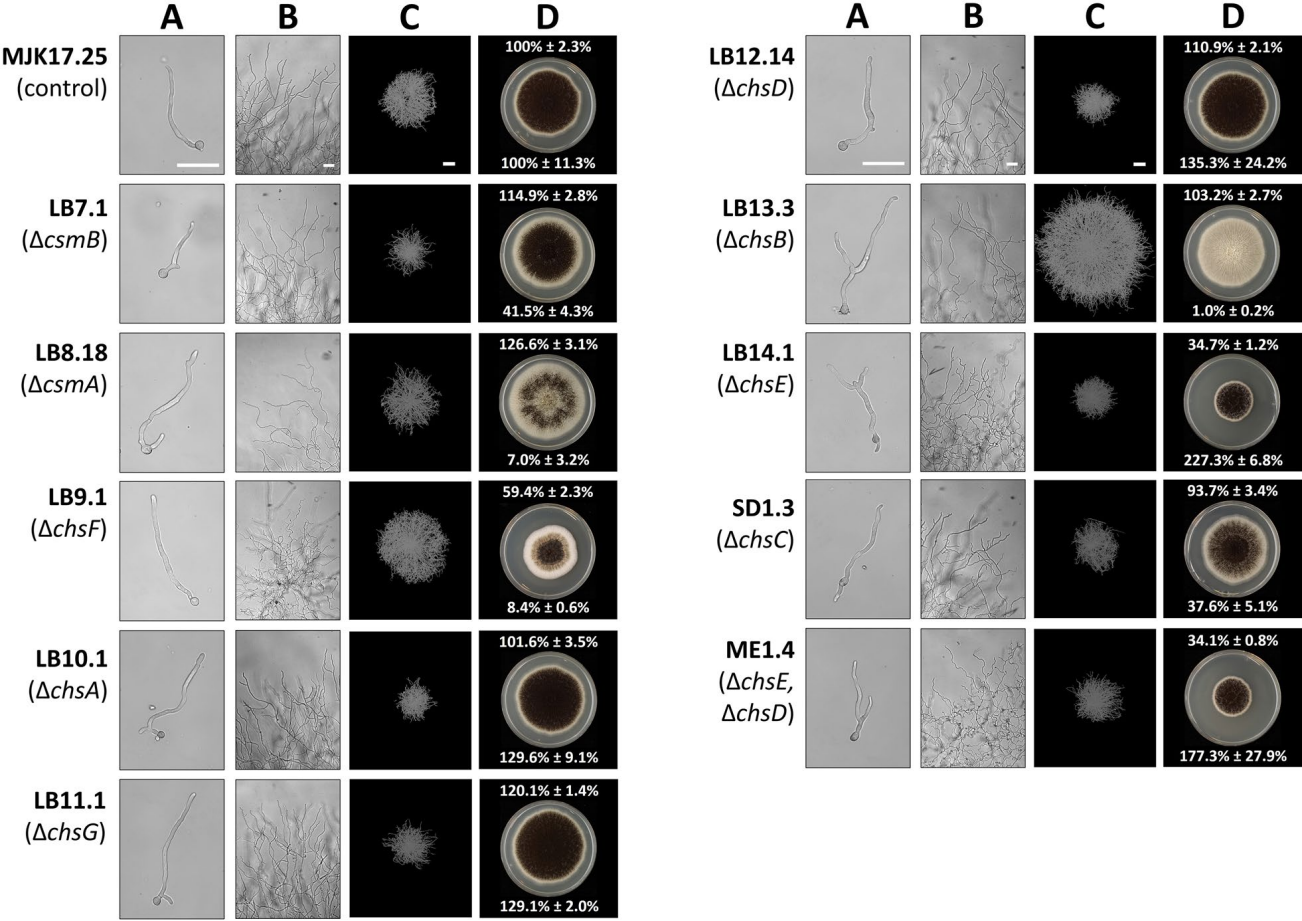
Deletion of chitin synthase encoding genes has diverse impacts on *A. niger* growth in solid and liquid cultivation. Light microscopic analysis of (**A**) germlings following 12 h incubation in CM liquid medium at 30°C without shaking or (**B**) young colonies following 26 h growth on MM agar at 30 ℃. Scale bar = 50 µm. Pellets grown in liquid culture were analysed as slices of 100 µm thickness using the µCT approach (**C**). Scale bar = 100 µm. Colony images (**D**) were captured following 6 days growth at 30 ℃ on MM. White values in upper section of each respective image are colony area normalized to the MJK17.25 control. White values in bottom of each colony image are spore titres normalized to colony area and reported as a percent of MJK17.25. Standard deviation from triplicate replicates is indicated

Cell wall synthesis is crucial for germination, whereby spores break dormancy, establish polarity, and begin polar hyphal extension [23]. We therefore speculated that chitin mutants might be defective in germination. Light microscopic analysis of germlings (12 h post inoculation) displayed no notable defects in rate of early hyphal extension during liquid growth (Fig. 2A). However, we did notice a clear impact of chitin synthase deletion in early mycelial networks relative to the control for Δ*csmB*, Δ*chsE* Δ*chsD*, and Δ*chsF*, with branching defects in young colonies on solid agar (Fig. 2B). We therefore conclude that individual chitin synthase encoding genes are dispensable for germination and germ tube polar extension, yet several begin impacting mycelial growth at early stages (< 26 h post inoculation of solid agar).

Colony growth and development on agar plates was impacted in all mutants (Fig. 2D), most strikingly in abundance of spores. When controlled for total colony area, spore titres were drastically reduced in strains Δ*csmA*, Δ*csmB,* Δ*chsB*, Δ*chsC*, Δ*chsE*, Δ*chsF* yet elevated in mutants Δ*chsA*, Δ*chsD*, Δ*chsG*, and Δ*chsE* Δ*chsD* (Fig. 2D). Thus, all chitin synthase genes are crucial mediators of colony spore titres. Additionally, we observed strong (Δ*chsE*, Δ*chsF,* Δ*chsE* Δ*chsD*) and moderately (Δ*chsC*) retarded radial growth rates. Interestingly, some mutants showed moderate increase in colony area, including Δ*csmA*, Δ*chsD,* Δ*chsG*. Strains Δ*csmA*, Δ*chsC* and Δ*chsF* also demonstrated colony sectoring. Taken together, this analysis demonstrates that all chitin synthase genes impact some aspect of *A. niger* growth or development, including mycelial growth, colony development, or spore titres.

### Septum chitin content is reduced in mutant isolates when grown on solid agar

**Fig. 3:**
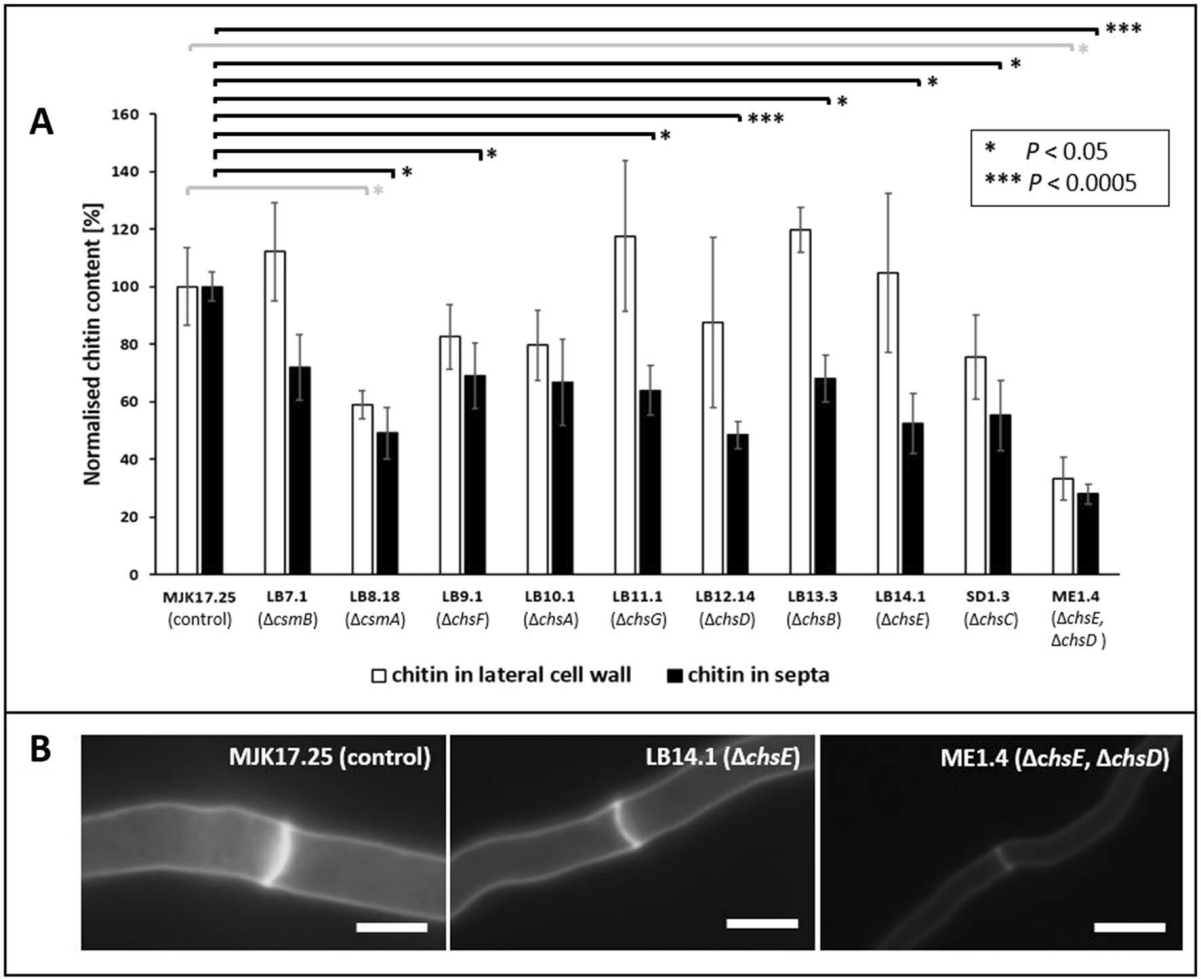
Fluorescent microscopic quantification of chitin in lateral cell wall and septa during growth on solid agar. Strains were grown on MM plates for 48 h at 20 ℃. Chitin was measured using Calcofluor White staining and fluorescent signal normalized to that of the control strain (**A**). Statistically significant changes relative to control are indicated (t-tests). Exemplar images are shown for MJK17.25 control and two mutants isolates (**B**). Scale bar = 5 µm

Next, we profiled cell wall chitin content in early colonies from minimal agar (48 h growth, 20 ℃, Fig. 3). Our rationale for using these growth conditions was (i) a complete absence of shear stress and (ii) the use of young colonies where we could easily analyse individual hyphae. In order to determine the difference between chitin content in septa and the lateral cell wall, we used Calcofluor White staining and fluorescent microscopy. In this assay, no mutant displayed statistically elevated chitin content in either septa or lateral wall relative to the control strain (Fig. 3). In contrast, both the Δ*csmA* and Δ*chsE* Δ*chsD* double mutant showed a highly reproducible reduction of chitin in the lateral cell wall (Fig. 3). Thus, we conclude that *csmA* encodes the chitin synthase that is principally responsible for chitin synthesis at this location. It is also interesting that *chsE* and *chsD* seem to play a functionally redundant role in lateral cell wall chitin synthesis, as evidenced by the reduction in chitin content in that was observed in exclusively in the double (and not single) mutants (Fig. 3). Interestingly, all mutants demonstrated marked reduction in chitin at the septa (although this was not statistically significant for Δ*csmB* or Δ*chsA*). This was surprising, given that phylogenetic and co-expression analysis suggested different evolutionary history and transcriptional deployment of these genes (Fig. 1, Additional file 1: Figures S2, S3 and S4). Thus, it seems that an important function of all *A. niger* chitin synthases is septal growth or maintenance.

### Cell wall composition drastically changes in mutant isolates during liquid growth conditions

**Fig. 4:**
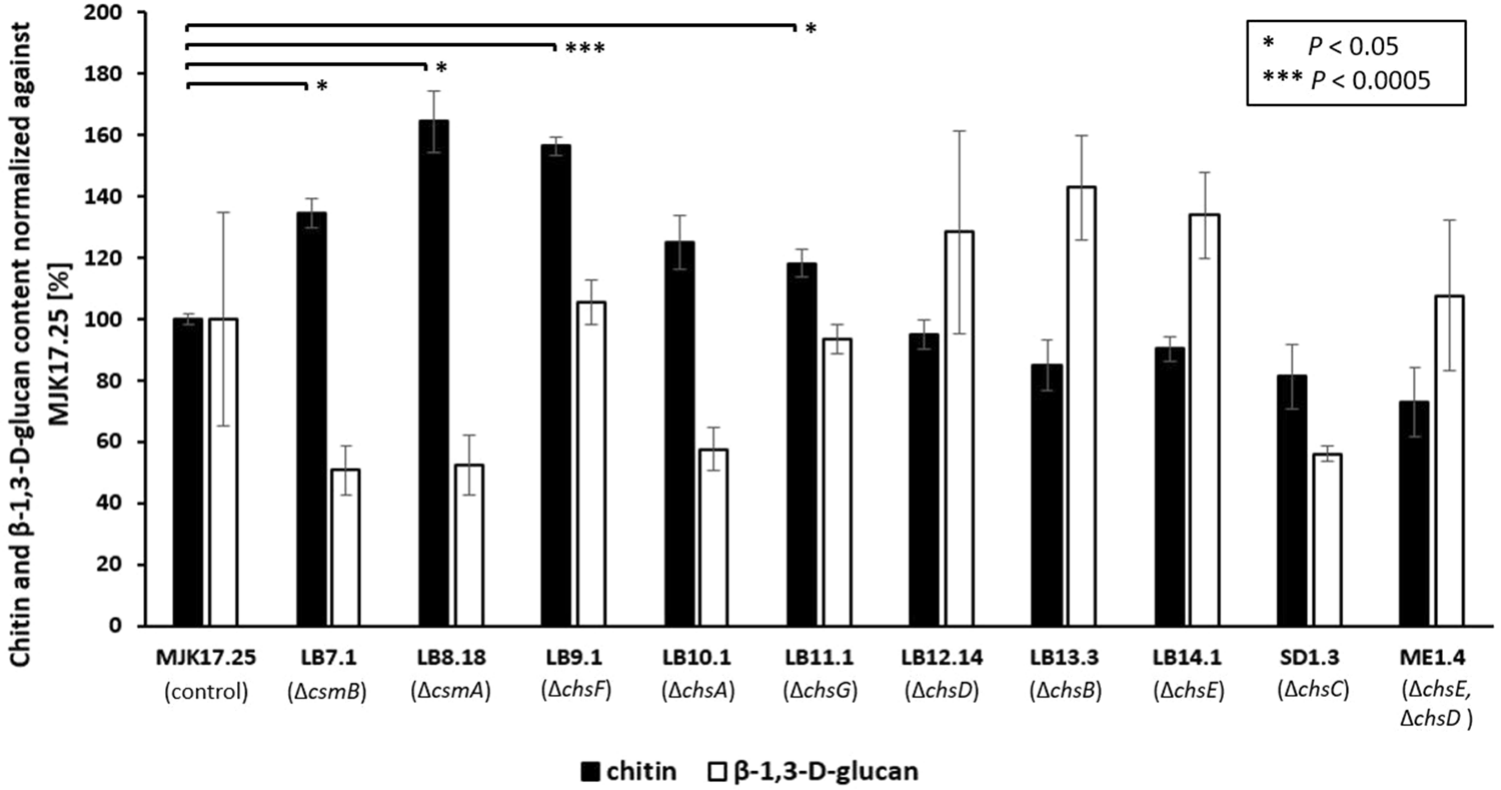
Quantification of chitin and 1,3-β-D-glucan in mutant strains during shake flask cultivation. Biomass was collected following 48 h growth in liquid CM (30 ℃, 250 RPM) and chitin or glucan content reported relative to the MJK17.25 control strain. Statistically significant changes relative to control are indicated (t-tests)

Most industrial applications of *A. niger* for protein, acid, or secondary metabolite production utilize submerged growth where high shear forces occur due to physical mixing of bioreactor media [14, 24]. Consequently, we wanted to analyse cell well content in this context. Given that fluorescent microscopic analysis of individual hyphae at late stage liquid fermentation is not possible due to pellet formation (Fig. 2C), we measured strain chitin and 1,3-β-D-glucan using standard hydrolysis assays. This revealed a significant increase in cell wall chitin content for mutants Δ*csmA*, Δ*csmB*, Δ*chsF*, and Δ*chsG* (Fig. 4). For mutants Δ*csmA* and Δ*csmB* this occurred alongside a notable- but not statistically significant- reduction in 1,3-β-D-glucan. Similar reduced levels of glucan relative to the control were also observed in Δ*chsA* and Δ*chsC* (Fig. 4). We speculate that elevated chitin in some mutants during liquid growth is possibly caused by overcompensation of remaining chitin synthases to reinforce the cell wall while under shear stress. It is interesting that in several cases, elevated chitin occurs alongside reduced glucan, indicating a substantial alteration in cell wall composition of mutants isolates under some growth conditions.

### Screening mutants under various cell wall perturbation reveals diverse phenotypic consequences of chitin synthase gene deletion

**Fig. 5:**
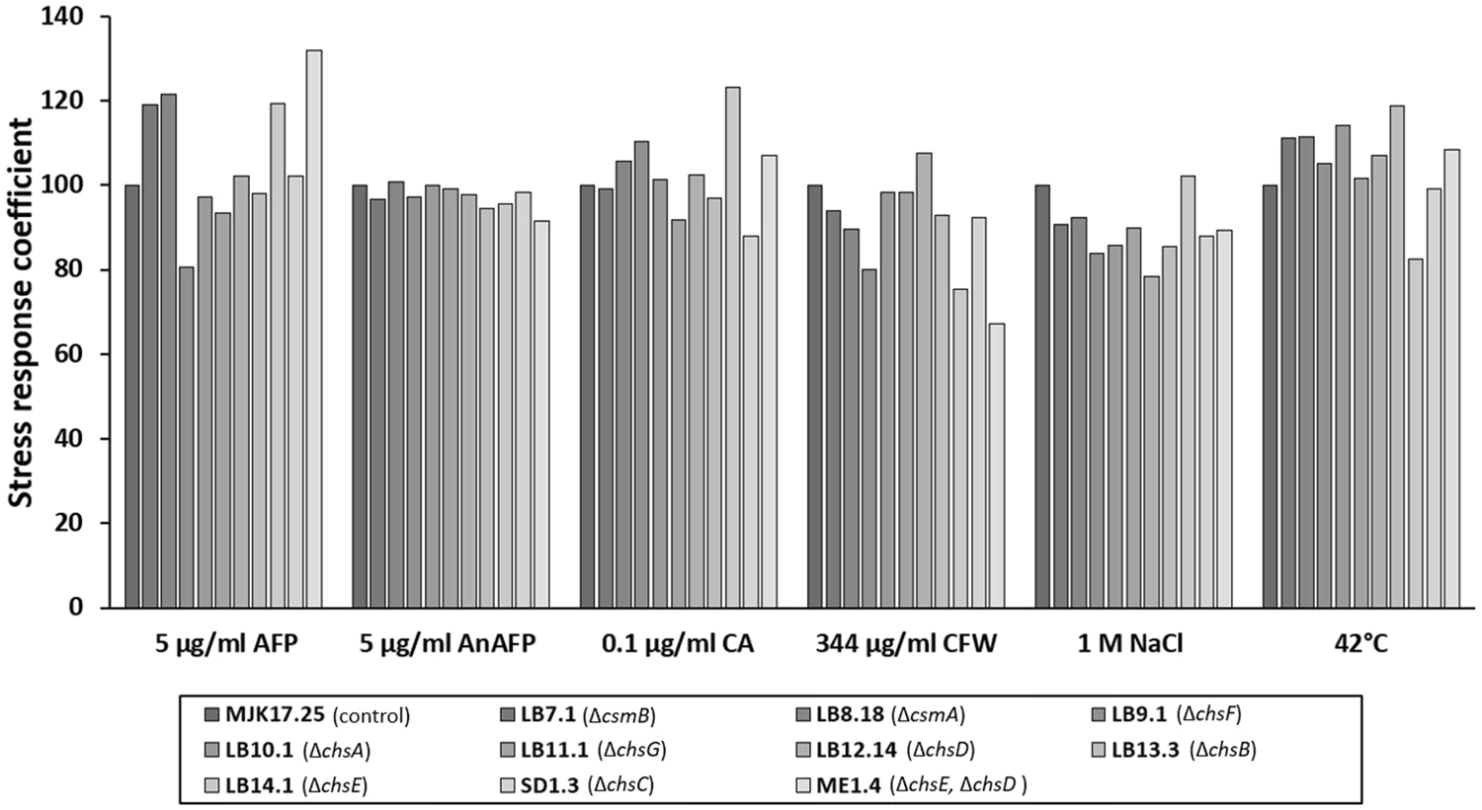
Stress response coefficients of mutants when challenged with a range of cell wall perturbation. Colony radial growth rates (6 days, 30 ℃ or 42 ℃) were determined from mutant or control strain on either MM or MM supplemented with indicated compound. Coefficients are calculated so that values > or < 100 indicate resistance or sensitivity respectively of mutants to indicated compound [25]. AFP- Antifungal protein. AnAFP- *A. niger* antifungal protein. CA- Caspofungin. CFW- Calcoflour white

Given the role of the cell wall in protection from the external environment [1–3], we hypothesised that mutants generated in this study would display sensitivity to various stress conditions. We thus generated stress response coefficients for respective mutants on agar plates as previously described ([25], Fig. 5). In this approach, radial growth on stress conditions is normalized to minimal media controls so that values > 100 or < 100 indicate resistance or susceptibility, respectively (see Methods). This assay included molecules that specifically bind and or perturb chitin (AFP [26], AnAFP [27], Calcofluor White), and glucan synthesis (caspofungin). Additionally, we grew strains under sublethal osmotic stress (1 M NaCl) and elevated temperature (42 ℃, [25, 27]). In general, no condition used in this assay caused universal sensitivity or resistance amongst the mutants (Fig. 5), an observation consistent with the hypothesis that chitin synthases play complex, non-redundant functions in *A. niger*. It should be noted, however, that with the exception of Δ*chsE*, growth on NaCl caused minor, yet detectable, sensitivity of the mutants relative to control, indicating most mutants are susceptible to osmotic challenge (Fig. 5). Other notable aspects of this screen were resistance (Δ*csmA*, Δ*csmB*, Δ*chsE*, and Δ*chsD* Δ*chsE*) and sensitivity (Δ*chsF*) to AFP. Such data support the hypothesis that AFP may specifically interact with the encoded enzymes or the chitin they synthesize. One concerning observation was the resistance of Δ*chsE* to sublethal caspofungin challenge, which indicates that mutations in this gene might lead to development of echinocandin resistance in Aspergilli. Finally, we could not robustly correlate any stress coefficients with colony development phenotypes (Fig. 2) septal/lateral cell wall chitin content (Fig. 3), or cell wall composition in liquid growth (Fig. 4), thus reinforcing the multifaceted and dynamic picture of chitin synthesis in *A. niger*.

### Chitin synthase encoding gene deletion impacts internal pellet structure in *A. niger*

**Fig. 6:**
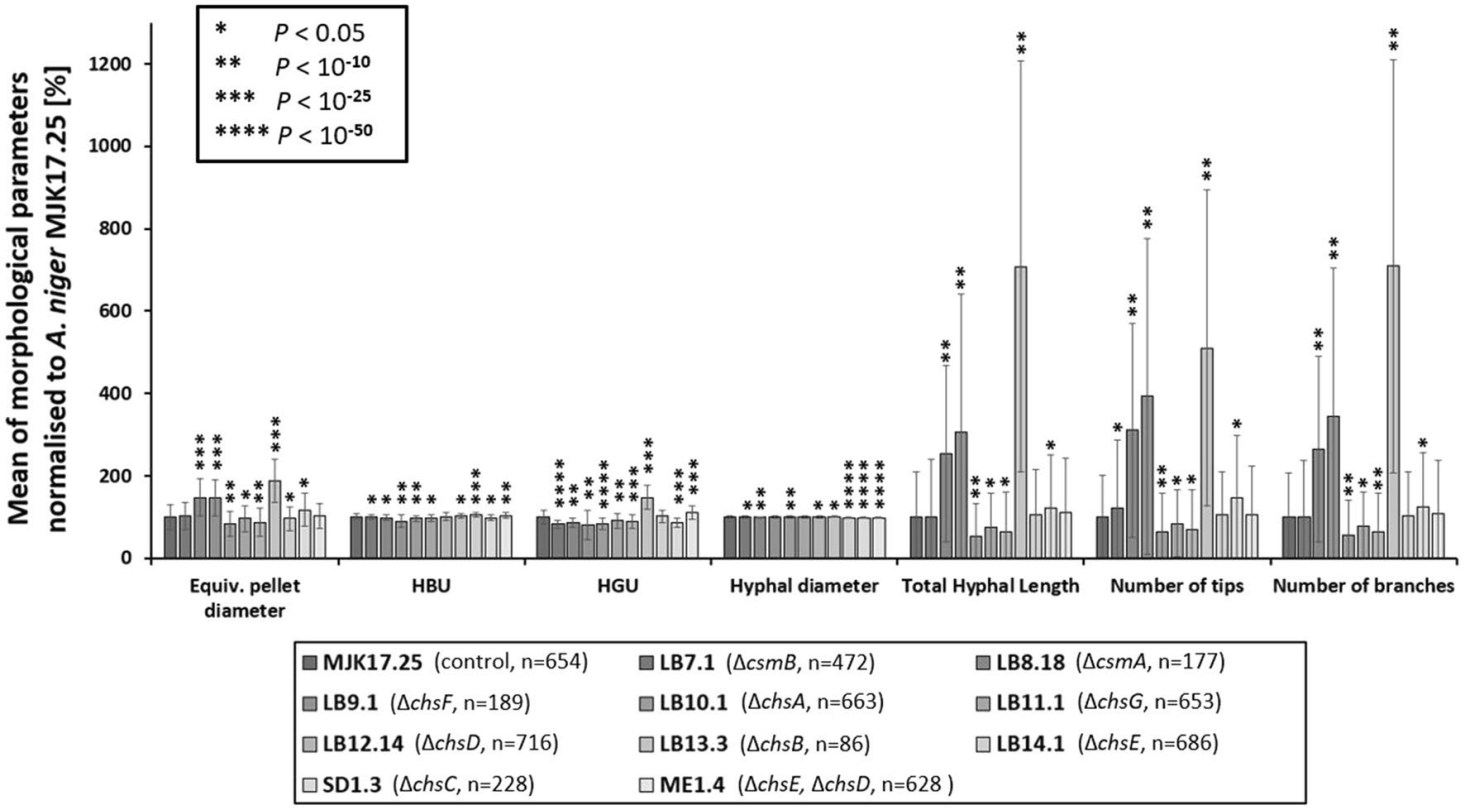
µCT quantification of pellet parameters during liquid cultivation. Isolates were growth in CM at 30 ℃, 250 RPM for 24 h. Number of pellets analysed per strain are indicated in parentheses. Equivalent pellet diameter, hyphal diameter, total hyphal length per pellet, number of tips and branches per pellet, hyphal growth unit (HGU, total hyphal length divided by number of tips) and hyphal branch unit (HBU, total hyphal length divided by number of branches) were calculated and reported relative to the control isolate. Statistically significant changes relative to control are indicated (t-tests)

**Fig. 7:**
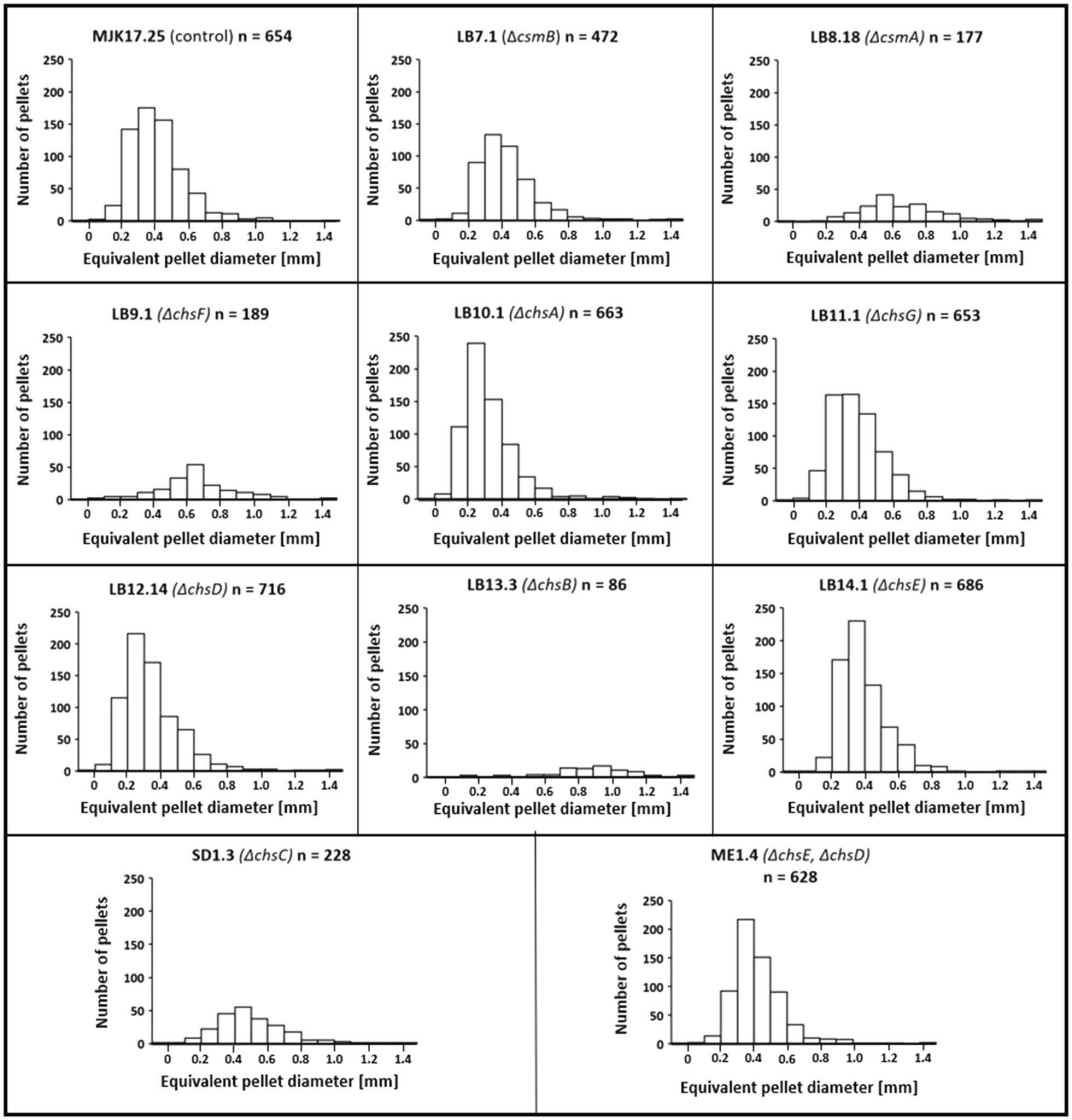
Heterogeneity of pellet diameter determined by µCT quantification. Pellet diameters are visualized using histograms. Number of pellets analysed per strain are indicated.

It is well established that macromorphological formation during submerged growth has critical implications for strain process engineering performance and product titres [14, 28]. We have recently developed a µCT approach that enables us to analyse fungal macromorphology in unprecedented detail, which notably includes quantification of hyphal growth inside the pellet core [29–31]. We hypothesized that chitin synthase gene deletion might impact various gross pellet parameters (e.g., diameter) and/or internal pellet architecture (e.g., hyphal branch rates). Using the µCT approach we could detect clear changes in submerged pellet sizes relative to the control strain in several mutants (Figs. 2C, 6 and 7). Most notably, Δ*chsB* produced large pellets (965 µm diameter) compared to the control (512 µm diameter), whereas Δ*chsA* produced the smallest pellets (422 µm diameter, Table 2). Taken together, these data demonstrate that deletion of chitin synthases is a viable strategy to control pellet size in *A. niger*.

**Table 2:** Quantitative analysis of submerged cultivations using µCT

	Equivalent pellet diameter [µm]		Hyphal diameter [µm]	Total hyphal length [m]	Number of tips [-]	Number of branches [-]	Hyphal branch unit (HBU) [µm]		Hyphal growth unit (HGU) [µm]	
MJK17.25 (control, n = 654)	512 ± 157		3.08 ± 0.06	0.37 ± 0.40	3,834 ± 3,830	2,267 ± 2,428	159 ± 11.1		90 ± 14.0	
LB7.1 (Δ*csmB*, n = 472)	528 ± 172		3.06 ± 0.06	0.36 ± 0.52	4,651 ± 6,305	2,247 ± 3,098	157 ± 9.6		74 ± 8.5	
LB8.18 (Δ*csmA*, n = 177)	753 ± 234		3.04 ± 0.05	0.92 ± 0.79	11,899 ± 9,959	5,983 ± 5,115	155 ± 11.8		77 ± 9.7	
LB9.1 (Δ*chsF*, n = 189)	750 ± 231		3.07 ± 0.11	1.12 ± 1.23	15,060 ± 14,708	7,784 ± 8,192	142 ± 22.8		73 ± 32.0	
LB10.1 (Δ*chsA*, n = 663)	422 ± 157		3.06 ± 0.06	0.19 ± 0.29	2,471 ± 3,539	1,262 ± 1,904	152 ± 12.0		75 ± 11.3	
LB11.1 (Δ*chsG*, n = 653)	490 ± 160		3.08 ± 0.08	0.28 ± 0.30	3,216 ± 3,078	1,769 ± 1,860	155 ± 13.5		82 ± 15.7	
LB12.14 (Δ*chsD*, n = 716)	445 ± 176		3.09 ± 0.09	0.23 ± 0.35	2,684 ± 3,644	1,438 ± 2,130	160 ± 15.2		80 ± 15.4	
LB13.3 (Δ*chsB*, n = 86)	965 ± 269		3.10 ± 0.05	2.59 ± 1.83	19,533 ± 14,700	16,074 ± 11,374	162 ± 7.4		132 ± 25.2	
LB14.1 (Δ*chsE*, n = 686)	491 ± 145		3.02 ± 0.04	0.38 ± 0.41	4,017 ± 4,057	2,294 ± 2,479	166 ± 8.6		91 ± 12.7	
SD1.3 (Δ*chsC*, n = 228)	601 ± 205		3.00 ± 0.04	0.45 ± 0.47	5,657 ± 5,763	2,848 ± 2,977	156 ± 11.0		77 ± 10.2	
ME1.4 (Δ*chsE* + Δ*chsD*, n = 628)	523 ± 156		3.02 ± 0.05	0.40 ± 0.48	4,008 ± 4,587	2,461 ± 2,906	164 ± 9.9		99 ± 14.7	

Mean values, standard deviation and number of pellets analysed per strain are given (n)

With regards to the pellet core, all mutants generated in this study were significantly impacted in various aspects of hyphal growth inside the pellet (Fig. 2C, Figs. 6 and 7). This included both increase and decrease in total hyphal length, branch rates, and tip numbers relative to MJK17.25 (Figs. 6 and 7 Table 2). It is remarkable that fungal pellets have substantial cumulative hyphal length, with the lowest (Δ*chsA*) and longest (Δ*chsB*) average total length 19 cm and 112 cm respectively (Table 2). Interestingly, this assay also detected statistically significant changes in hyphal diameter for eight of the mutants when compared to control (Figs. 6 and 7, Table 2), although in each instance this change was very minor e.g., 0.02–0.04 µm (Table 2). We therefore conclude that chitin and cell wall structure in general are crucial determinants of hyphal shape and branching in the pellet core.

### Deletion of chitin synthase genes is a viable approach to increase total secreted protein in *A. niger*

**Fig. 8:**
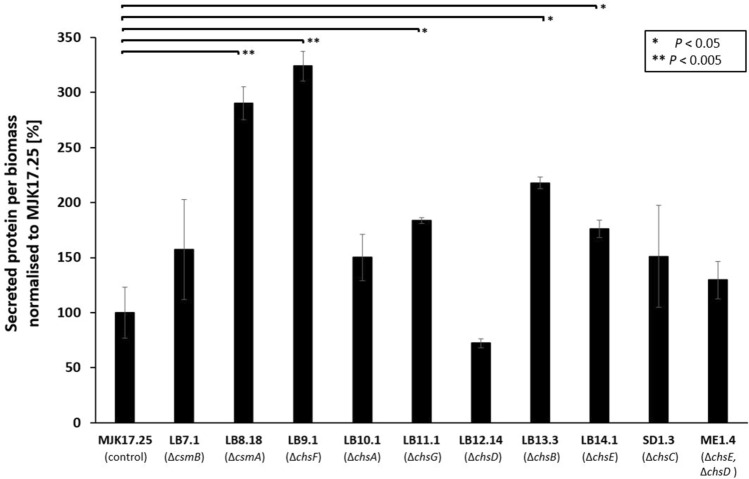
Total protein in culture supernatant. Strains were grown in CM media and incubated for 24 h (30°, 250 RPM). Secreted protein was determined using Bradford quantification and normalized to strain biomass (g). Values are reported as percentage relative to the control strain, with standard deviation from triplicate shake flasks reported. Statistically significant changes relative to control are indicated (t-tests)

**Fig. 9:**
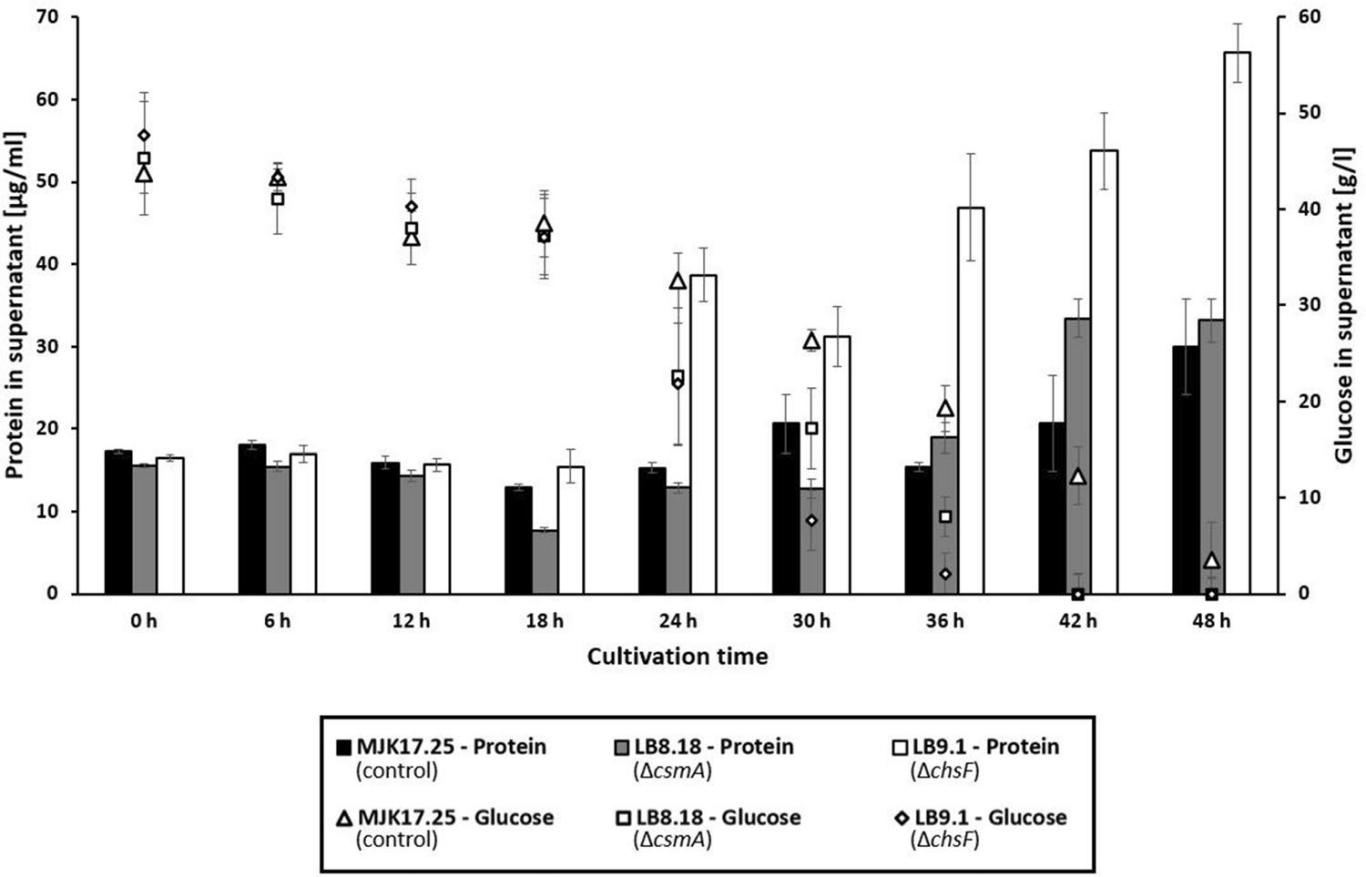
Glucose and total protein in culture supernatant throughout a time series of growth. Mutant isolates Δ*csmA* (LB8.18) and Δ*chsF* (LB9.1) and control strain were cultured for 48 h in liquid CM (5 % glucose, 30°, 250 RPM) and supernatant total protein (left axis) and glucose (right axis) determined at indicated time points

In order to test if the mutants generated in this study have possible biotechnological applications, we measured total protein in culture supernatant following 24 h growth in standard shake flask models of fermentation. This demonstrated Δ*csmA*, Δ*chsB*, Δ*chsE*, Δ*chsF*, and Δ*chsG* had elevated titres of protein in culture supernatant compared to the control strain (Fig. 8). It should be noted that this increase was substantial for mutants Δ*csmA* and Δ*chsF*, which reported approximately three-fold increase relative to isolate MJK17.25 (Table 3). In order to further test the utility of these isolates as protein production strains, Δ*csmA* and Δ*chsF* were taken into a second screen whereby (i) glucose was increased to 5% to put further pressure on the secretory system (ii) cultivation time was extended to 48 h to test protein secretion at later stages, (iii) total protein was measured every 6 h and (iv) we quantified glucose consumption as a second measurement of fungal growth stage (Fig. 9). Under these conditions, mutant Δ*csmA* generated comparable protein secretion with the control strain, whereas significantly more protein was found in culture supernatant of Δ*chsF*. Importantly, at 36 h post inoculation during exponential growth, Δ*chsF* produced more than three times the control (Fig. 9). Taken together, deletion of *A. niger chsF* is a highly promising lead for engineering protein hyper-production strains.

**Table 3:** Total protein in culture supernatant

	Protein per biomass [mg/*g*]	Protein per culture supernatant [mg/l]
MJK17.25 (control)	1.55 ± 0.44	11.49 ± 3.20
LB7.1 (Δ*csmB*)	2.45 ± 0.86	13.75 ± 3.07
LB8.18 (Δ*csmA*)	4.51 ± 0.29	29.39 ± 2.11
LB9.1 (Δ*chsF*)	5.03 ± 0.26	31.29 ± 1.27
LB10.1 (Δ*chsA*)	2.34 ± 0.40	17.56 ± 1.66
LB11.1 (Δ*chsG*)	2.86 ± 0.05	20.98 ± 0.90
LB12.14 (Δ*chsD*)	1.12 ± 0.08	8.72 ± 0.96
LB13.3 (Δ*chsB*)	3.39 ± 0.10	24.03 ± 2.02
LB14.1 (Δ*chsE*)	2.74 ± 0.15	20.93 ± 0.90
SD1.3 (Δ*chsC*)	2.35 ± 0.88	16.02 ± 5.41
ME1.4 (Δ*chsE* + Δ*chsD*)	2.02 ± 0.32	11.44 ± 0.71

Mean values and standard deviation from triplicate replicates are given

## Discussion

As a key structural component of the fungal cell wall, chitin plays a multifaceted role in fungal growth, cell shape, and protection from the environment [1–3]. Despite interest in chitin synthase encoding genes from medical, plant, and industrial mycologists, studies that probe all predicted chitin synthase ORFs for a given fungus are rare (especially in filamentous fungi). It should be noted that other works have targeted individual chitin synthase encoding genes in *A. niger* and other Aspergilli. For example, RNAi based knock-down of what the authors name *chsC* (which we assume denotes gene An14g00660) caused reduced sporulation and elevated titres of citric acid during shake flask culture [32]. Most chitin synthase functional analyses have been conducted in the model system *A. nidulans* [33], which demonstrated, for example, defective hyphal polarity when *csmA* and *csmB* were deleted [34], reduced sporulation following *chsA* deletion [35], hyper-branching in a *chsB* mutant [36], and no detectable impacts of *chsC* targeting [37]. Localisation studies (e.g., using GFP tagged proteins) have also provide clues with regards to the role of individual synthases. For example, ChsB in *A. nidulans* localizes to germ tube tips, the hyphal apex, nascent septa, and between metulae and phialides, strongly indicating this protein mediates chitin synthesis during early growth, mature hyphal extension, and asexual development [38]. Despite these functional insights, comprehensive analysis of all mutants in a single work has not been done to date.

**Fig. 10:**
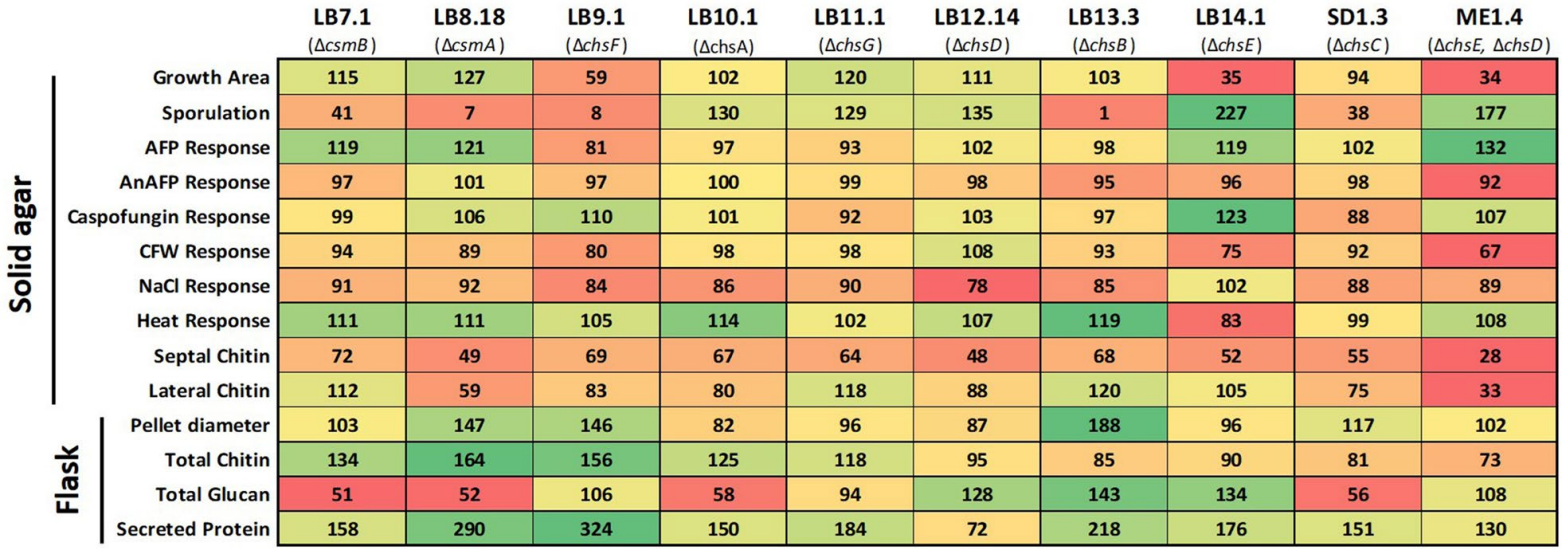
Quantitative summary of strain phenotypes in liquid and solid agar throughout this study. All values are reported as a percentage of the MJK17.25 control, with elevated or reduced values shown in green and red, respectively

This study has thus conducted the first systemic functional analysis of the chitin synthase gene repertoire in the multipurpose cell factory *A. niger.* Phylogenetic analysis suggests the nine predicted chitin synthase genes in *A. niger* can be assigned to three groups with common ancestors (Fig. 1). Transcriptional analysis and co-expression network construction predicted largely disparate expression profiles between these genes, suggesting they probably play different roles in this fungus (Additional file 1: Figures S3 and S4). Gene deletion had a wide variety of impacts (summarised in Fig. 10), which in general supported various functions for *A. niger* chitin synthases. These data demonstrated that all strains (i) germinated (ii) had spore titres different from the control (iii) display reduced chitin at the septa and (iv) had modified pellet cores when grown in liquid culture. Thus, despite the many strain specific phenotypes reported in this study, our data support the notion that all nine *A. niger* chitin synthase encoding genes are dispensable for cell wall synthesis during early germ tube extension, and play non-redundant roles in chitin synthesis at septa, sporulation, and the development of fungal pellets in submerged cultivation. It should be noted that the true extent of functional redundancy for *A. niger* chitin synthase encoding genes requires generation of more double mutant isolates. Additionally, while we have quantified chitin, we did not profile variation of mutant cell wall organisation, which could also explain some of the observed phenotypes.

This study has also enabled us to assign specific roles to given genes and cognate enzymes (Fig. 10), including those impacting radial colony growth rates (ChsE, ChsF), chitin content in the lateral cell wall (CsmA), putative chemical genetic interactions with AFP (CsmA, CsmB, ChsE*,* ChsF), resistance to echinocandins (ChsE), those for impacting pellet diameter (ChsA, ChsB), and a high-priority lead for maximizing protein titres during fermentation (ChsF).

From a methodological perspective, several insights from this study might be useful for future systematic analyses, either for genes encoding other *A. niger* cell wall biosynthetic enzymes or chitin synthases in other fungi. Firstly, *chsF* clearly demonstrates that low and/or rare expression of a gene should not result in exclusion from functional analysis. This ORF was rarely transcriptionally active, yet ultimately proved to be the most promising from applied perspectives. The precise explanation for this is puzzling- possibly, the transcriptional conditions in the meta-analysis simply did not capture the instances where *chsF* is expressed, although given this includes 155 conditions it seems unlikely [18]. An alternative possibility is that sensitivity of the microarray platform is insufficient for a synthase that is expressed, but at low levels. Recent advances in sensitivity i.e., RNA-seq, should soon test this hypothesis [39]. In any event, it is clear that transcriptional resources can guide time and labour-intensive deletion experiments, but this should ideally not be used to exclude genes that are identified by genomics or phylogenetics (e.g., Fig. 1).

A second methodological insight was gained from quantifying chitin in two disparate growth conditions, specifically solid agar (Fig. 3) and in high shear liquid cultivation (Fig. 4). Our data reinforce the notion that analysis of fungi under liquid growth (which is normally combined with shaking or stirring) should take into consideration the activation of cell wall stress and subsequent remodelling. Interestingly, *csmA* deletion caused reduced chitin in lateral cell wall and septa on solid agar (Fig. 3) yet a significant increase in total chitin when shaken in liquid (Fig. 4). Our explanation is that CsmA is the major chitin synthase in *A. niger*, and deletion of this gene causes strong chitin defects in the cell wall which results in over-compensation by other synthases following activation of cell wall integrity. Thus, our study shows fungal cell wall mutants should be analysed in both solid and submerged cultivation for a more complete understanding of null strain phenotypes.

A final technical observation from this work is that quantitative analysis of the pellet core revealed multiple impacts of gene deletion that would not be detected from analysis of gross macromorphological structure (e.g., pellet diameter, aspect ratio, surface structure, Fig. 6). Thus, while previous analyses from our group and others have focused on such measurements [20, 40], it should not be overlooked that two or more pellets with similar macromorphologies can have a very different core architecture. This certainly plays a crucial role in diffusion of oxygen and nutrients [31, 41], potentially resulting in altered production rates. Thus, methods for measuring internal pellet architecture that we have developed [31, 41] must be more commonly utilized and optimized in terms of throughput.

From a biotechnological perspective, we did not find any notable correlations between mutant submerged extracellular protein titres and any of the phenotypes measured in this study (Fig. 10). However, our data demonstrate increased titres of extracellular protein following deletion of *chsF* (Fig. 9). Encouragingly, this was consistent with our previous work which demonstrated *A. niger* isolates that were sensitive to cell wall stress via chitin perturbation also tended to produce more total protein in shake flasks [16]. Given that *A. niger* is used to produce multiple enzymes worth several billion euros per year [24], deletion of *chsF* is a promising lead that could enable more energy efficient and cost-effective protein fermentation for large scale industries. One possible drawback of this strain is that sporulation was reduced on solid agar (Fig. 2), although this was not sufficiently defective as to prevent preparation of spore inoculums. A precise mechanism for elevated protein titres in supernatant during Δ*chsF* liquid culture was beyond the scope of this study, yet we suggest this could be due to (i) increased ability of protein to transverse a chitin deficient cell wall, (ii) an as yet unknown intracellular response to shear stress causing increased extracellular protein as cells lyse, or (iii) modifications to the pellet core which enable increased diffusion of nutrients/oxygen to areas that would otherwise become starved or hypoxic, respectively. Given that *chsF* is very rarely expressed (and at low levels), we consider it unlikely that elevated protein titres in the respective mutant can be mechanistically explained whereby the absence of ChsF in vesicles allows delivery of more extracellular proteins through the classical secretory system. Future studies from our group will address the mechanistic explanation of these data in *A. niger* and other fungal cell factories.

## Conclusion

In this study we have conducted the first analysis of the full chitin synthase encoding gene repertoire in the multipurpose cell factory *A. niger.* This has revealed common roles of chitin synthases; on solid agar, gene deletion in each respective mutant impacted spore titres and septa, and in liquid growth, pellet core structure was changed. Various gene specific observations have also been revealed in this study, including synthases that impact colony radial growth rates, hyphal cell wall composition, response to abiotic perturbation, amongst others. Most interestingly from an industrial microbiological viewpoint, *chsF* deletion increases extracellular total protein in shake flask supernatant when compared to the control isolate. From a methodological perspective, µCT quantification of multiple mutants in parallel provides proof of principle that this analysis can be applied to understand the consequences of fungal genetic manipulation in unprecedented detail.

### Methods

## Phylogenetic analysis

We used fungiDB [42] to search fungal genomes for predicted chitin synthase encoding genes using Enzyme Commission number 2.4.1.16 (chitin synthase). *D. melanogaster* chitin synthases were identified from InsectBase using the same approach [43]. All phylogenetic analysis and sequence alignment were conducted the "build" function of ETE3 v3.1.1 as implemented on the web resource GenomeNet (https://www.genome.jp/tools/ete/). All analysis parameters were kept as default, with the exception of ‘pairwise alignment’ which was adjusted to ‘slow/accurate’. The phylogenetic tree was constructed using fasttree [44]. Orthologs of *A. niger* chitin synthase encoding genes were found using FungiDB’s “orthology and synteny” search tool using default parameters.

### Co-expression network analysis

Respective co-expression networks were generated for each predicted chitin synthase encoding gene from the publicly available dataset on fungiDB [42] as previously described [19–21]. Spearman correlation threshold was set to a minimum of ≥ 0.5, whereby any gene with a transcriptional signature similar that of the target chitin synthase gene was included in the network. GO enrichment of individual networks was conducted using fungiDB, with Benjamini–Hochberg false discovery correction applied.

### Microbial strains and cultivation conditions

Fungal strains used or generated in this study are given in Table 1. Mutants were constructed using the well-established *pyrG* marker in uridine auxotrophic strain MA169.4 ([22], Table 1). Note, the MA169.4 derivative strain MJK17.25- whereby the *pyrG* marker is restored- was used as the appropriate control isolate throughout this study (Table 1). All cloning and plasmid propagation were conducted using *Escherichia coli* DH5α using ampicillin as selection. Fungal propagation used standard minimal and complete media where indicated. *A. niger* was routinely grown at 30°C unless otherwise stated.

### Molecular techniques

All plasmids, primers, and detailed cloning information conducted in this study are as described previously [45]. *A. niger* transformation was conducted using a PEG-protoplast approach. Briefly, all strains with the exception of SD1.3 were made using *AopyrG* split marker cassettes. Genome editing to generate mutant SD1.3 followed a previously described approach, with MA169.4 progenitor strain transformed with two sgRNA plasmids targeting loci inside the *chsC* ORF, a Cas9 encoding plasmid, and a linear *AopyrG* encoding cassette for targeted gene replacement [45]. Gene deletion in all mutants was confirmed using various PCR analysis for targeted integration of the *AopyrG* marker at the desired locus. Additionally, deletion of the target ORF was confirmed- and of ectopic cassette integration ruled out- using standard Southern blot (Additional file 1: Figure S6).

### Quantitative analysis of colony spore titres and growth coefficients on solid agar

Spore titres were normalised to colony area in triplicate using the following approach. 1000 spores in 10 µl sterile saline were inoculated onto solid complete media and incubated for 6 days. Colony images were captured after which all spores were harvested. Titres were determine using a haemocytometer and spore density normalised to colony area.

*A. niger* growth coefficients were calculated in duplicate according to the published protocol [25]. The indicated concentrations of each molecule were selected based on a pre-screen with control isolate MJK17.25 that was used to identify sublethal stress (i.e., 5–50% reduction in colony radial growth) relative to minimal media only controls (Additional file 1: Figure S7). For the full screen, minimal media control or minimal media supplemented with indicated concentration of respective compounds were inoculated at the plate centre with 1000 spores in 10 µl sterile saline. Plates were incubated for 6 days after which radial growth rates were measured, and growth coefficients calculated as previously described [25], so that that values > or < 100 indicate resistance or sensitivity respectively of mutants to indicated compound. Average values from duplicate replicates are shown.

### Quantitative fluorescent microscopy

For quantification of chitin content in *A. niger* lateral cell wall or septa, spores were dotted onto minimal agar with a cotton stick and incubated for 48 h at 20 ℃. For staining, 10 µl Calcofluor White stain (Sigma-Aldrich Corporation, USA) was added followed by 10 µl 10% (w/v) potassium hydroxide. Images were captured using a Leica DM5000B-CS microscope (Leica Mircosystems GmbH, Germany) in fluorescence mode equipped with CFP filter cube, a Leica DFC365FX camera (Leica Mircosystems GmbH, Germany) and LasX software (LeicaMircosystems GmbH, Germany). Intensity values of lateral cell wall or septa were measured in triplicate per hyphae, with background signal subtracted. A minimum of three hyphae were analysed per strain, with triplicate values analysed per hyphae.

### Analysis of cell wall composition

β-1,3-D-glucan was quantified in biomass from submerged cultivation as previously described [46]. Fungal material was harvested by filtration, washed with sterile water, and lyophilized. Biomass was ground with a pestle and mortar before 5 mg (± 0.5 mg) was washed with 0.1 M NaOH, centrifuged and supernatant discarded before resuspension in 250 µl 1 M NaOH. Sample was then ultrasonicated for 1 h, centrifuged, and the glucan containing supernatant diluted 1:20 in 1 M NaOH. Concentration of glucans were quantified in technical duplicate with Anilin Blue and curdlan used as a standard using a FLUOstar Omegaplate reader (BMG Labtech GmbH, Germany) and excitation wavelength of 355 nm and emission wavelength of 520 nm.

Chitin was quantified from biomass prepared, lyophilized, and ground as described [45]. Cell wall was extracted from ~ 50 mg freeze dried biomass by two washes in 1 M NaCl and a single wash in distilled water with centrifugation and supernatant removal between each step. Samples were resuspended in cell wall extraction buffer (0.2% SDS, 5 mM EDTA, 100 mM β-mercaptoethanol) and boiled for 5 min, after which pellets were centrifuged and freeze dried. Next, 4 mg (± 0.5 mg) were autoclaved in 6 M HCl which was then evaporated at 75 ℃. Dried samples were beaten with glass beads for 20 min, centrifuged, and supernatant analysed by incubation with 0.75 M Na_2_CO_3_ and 4 % (v/v) acetylacetone solution and p-dimethylaminobenzaldehyde [45]. Absorbance was measured at a wavelength of 490 nm using a GloMax^®^-Multi Detection System (Promegacorporation, USA). Glucosamine was used as standard.

### µCT analysis of fungal pellets

Pellets were isolated from liquid culture following 24 h in complete media (30 ℃, 250 RPM). Pellets were gently washed in sterile water repeatedly to remove residual growth media, after which they were floated in 1 ml sterile water, snap frozen in liquid nitrogen and freeze dried overnight. 3D imaging was conducted at the Deutsches Elektronen Synchrotron (DESY) using the microtomography beamline P05 of PETRA III in Hamburg, Germany as previously described [29–31]. For quantification, images were analysed as previously described [47].

### Shake flask models of fermentation

Glucose concentration was determined using a kit (GLUCOSE GOD/PAP FLUID, mti-diagnostics GmbH, Germany) with sample absorbance determined at a wavelength of 560 nm using a GloMax^®^ Multi Detection System (Promega corporation, USA). Total extracellular protein was quantified in a minimum of technical duplicate using a Bradford assay, which was normalised to biomass.

### Supplementary Information

**Additional file 1: Figure S1.** Schematic representation of protein domains in nine chitin synthase encoding genes predicted in *A. niger*. **Figure S2.** Expression of chitin synthase encoding genes across 155 conditions. Data were extracted from previous microarray analysis [18]. **Figure S3.** Word cloud depicting enriched GO terms (Biological Process) from chitin synthase encoding gene co-expression networks. Data were extracted from previous co-expression analysis [18]. **Figure S4.** Word cloud depicting enriched GO terms (Cellular Component) from chitin synthase encoding gene co-expression networks. Data were extracted from previous co-expression analysis [18]. **Figure S5.** Spearman correlation coefficients between chitin synthase encoding genes. Correlations >0.5 are shown. Data were extracted from previous co-expression analysis [18]. **Figure S6.** Southern blot confirmation of single cassette integration for *A. niger* transformants passing PCR quality control. **Figure S7.** Normalised radial colony growth rates of *A. niger* mutants on solid agar. 1000 spores were spotted onto MM agar +/- the indicated stress condition and incubated for 6 days at either 30 ℃ or 42 ℃. Colony diameters were measured and normalized to the MJK17.25 control at the respective condition. **Table S1.** Chitin synthase encoding genes predicted in *A. niger* CBS 513.88 assigned to various enzyme classes. The abbreviation recCHS describes a recombined chitin synthase. **Table S2.** Orthologues for *A. niger* chitin synthase encoding genes amongst indicated Aspergilli. ORF code, gene name and amino acid sequence homology compared to *A. niger* CBS 513.88 are listed.

## Data Availability

The data sets, strains used and/or analysed during the current study, and sequences are available from the corresponding authors on reasonable request.
