# Supplementary figures and images for: Breaking down barriers: comprehensive functional analysis of the Aspergillus niger chitin synthase repertoire
Source: Fungal Biol Biotechnol. 2024 Mar 11;11:3. doi: 10.1186/s40694-024-00172-7 (PMC10926633; doi:10.1186/s40694-024-00172-7)

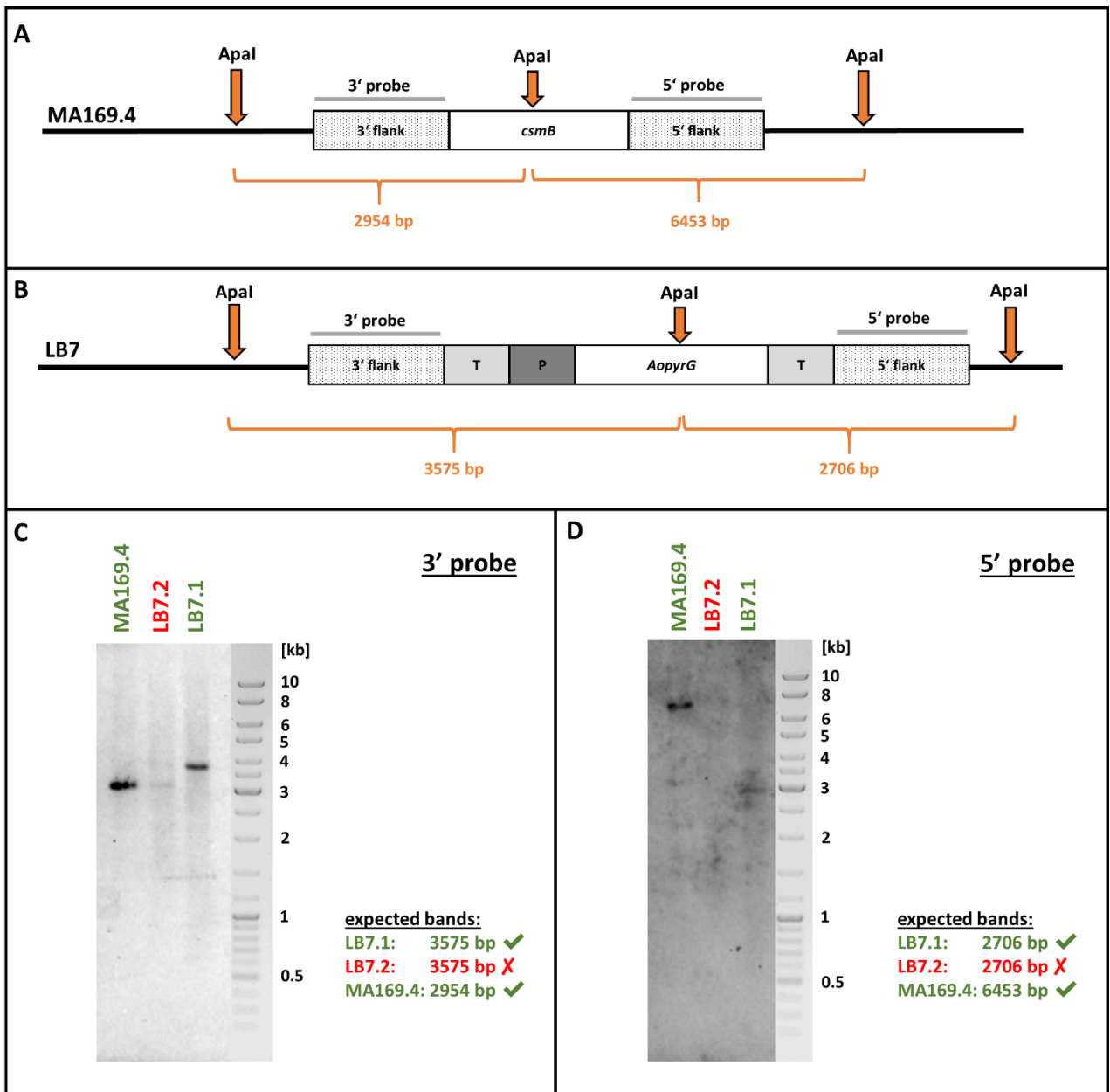

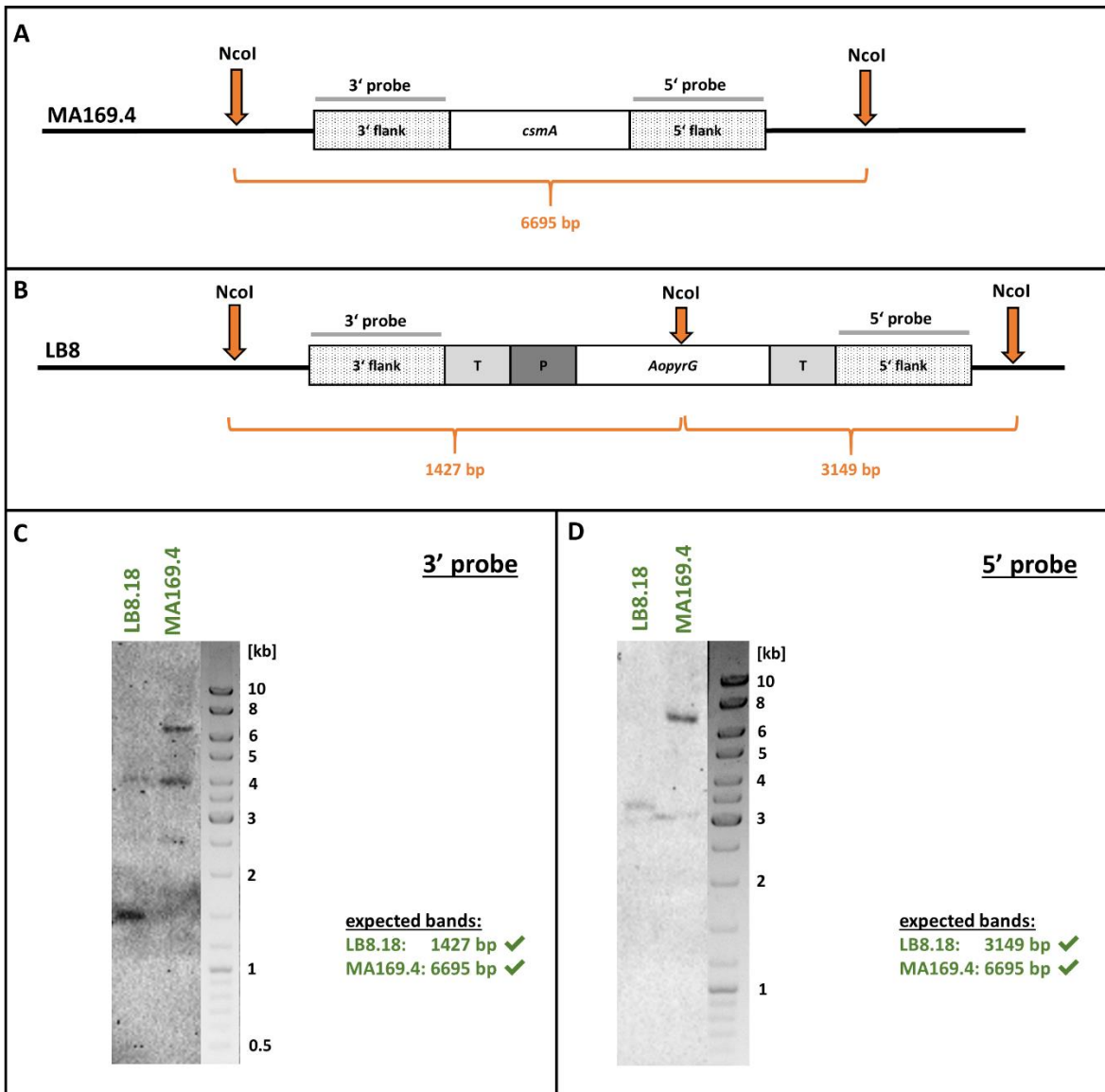

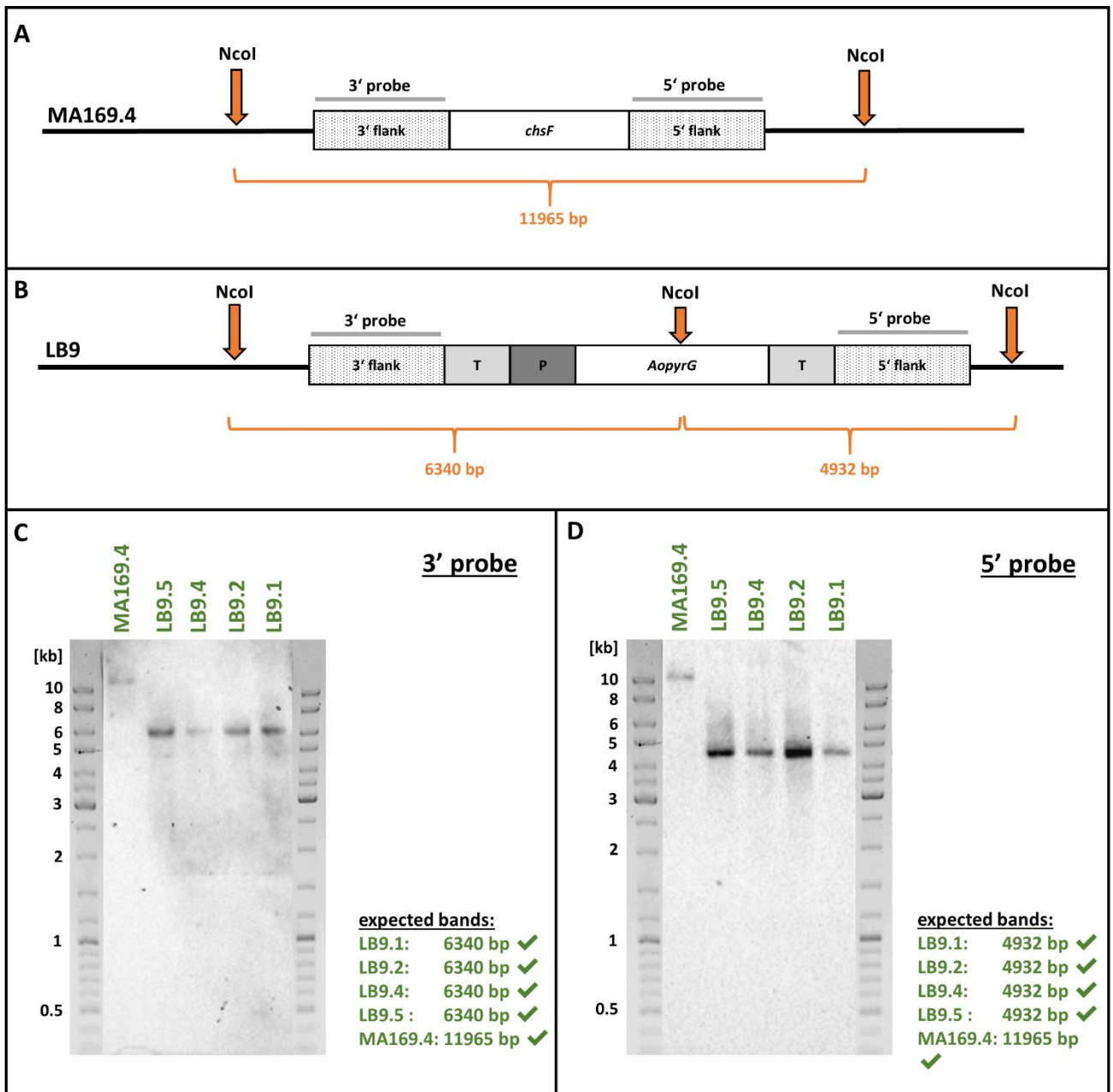

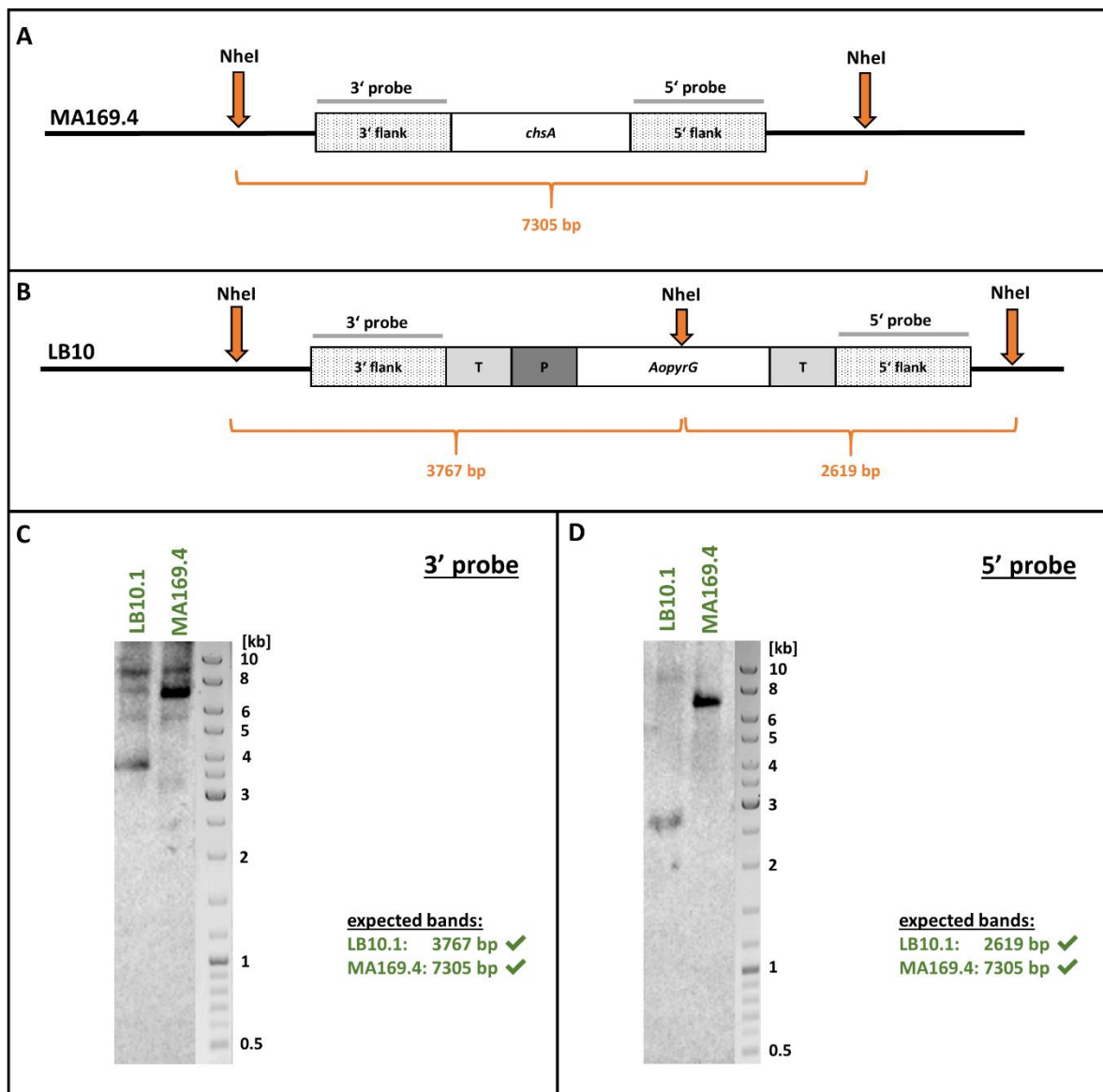

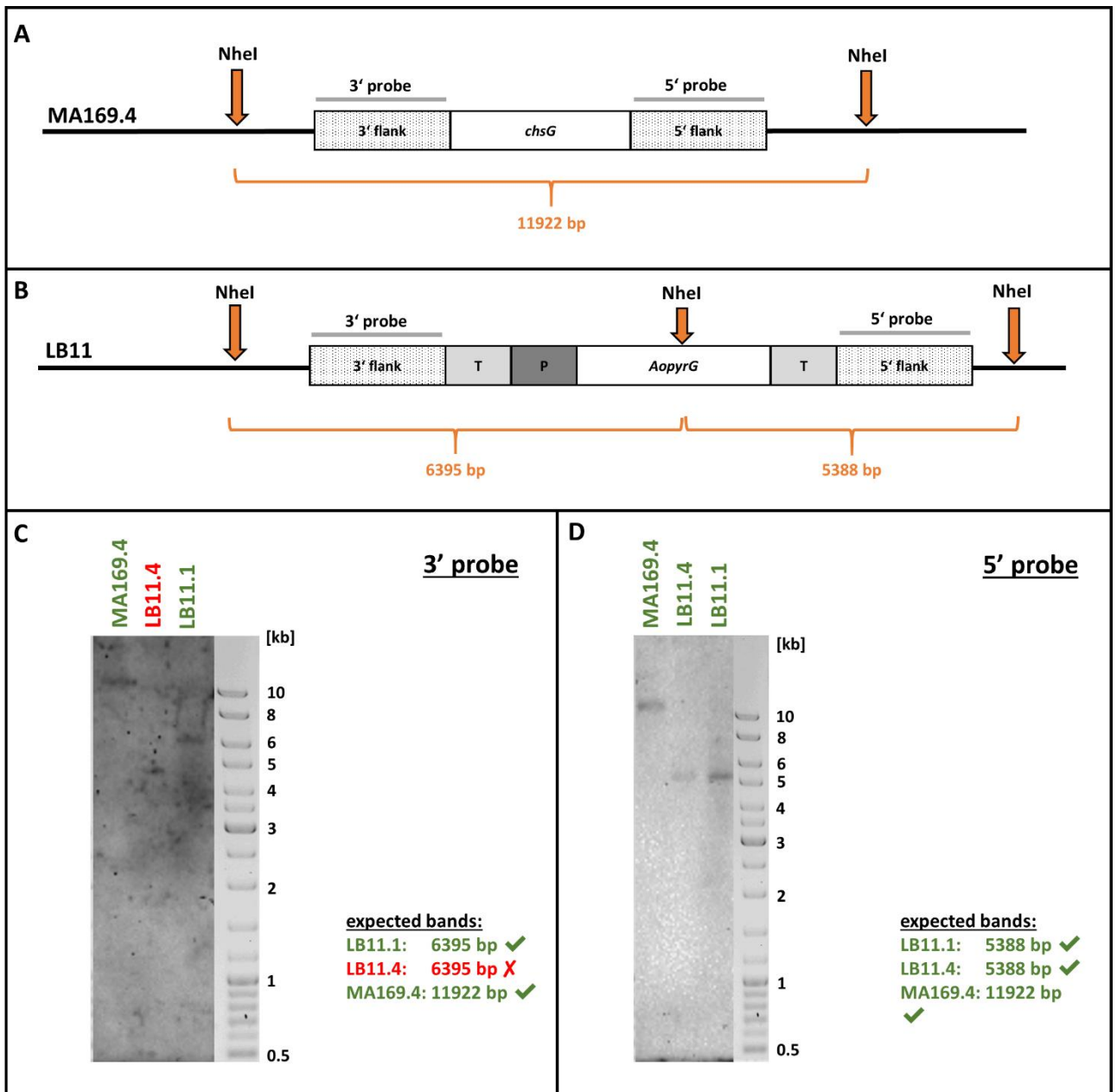

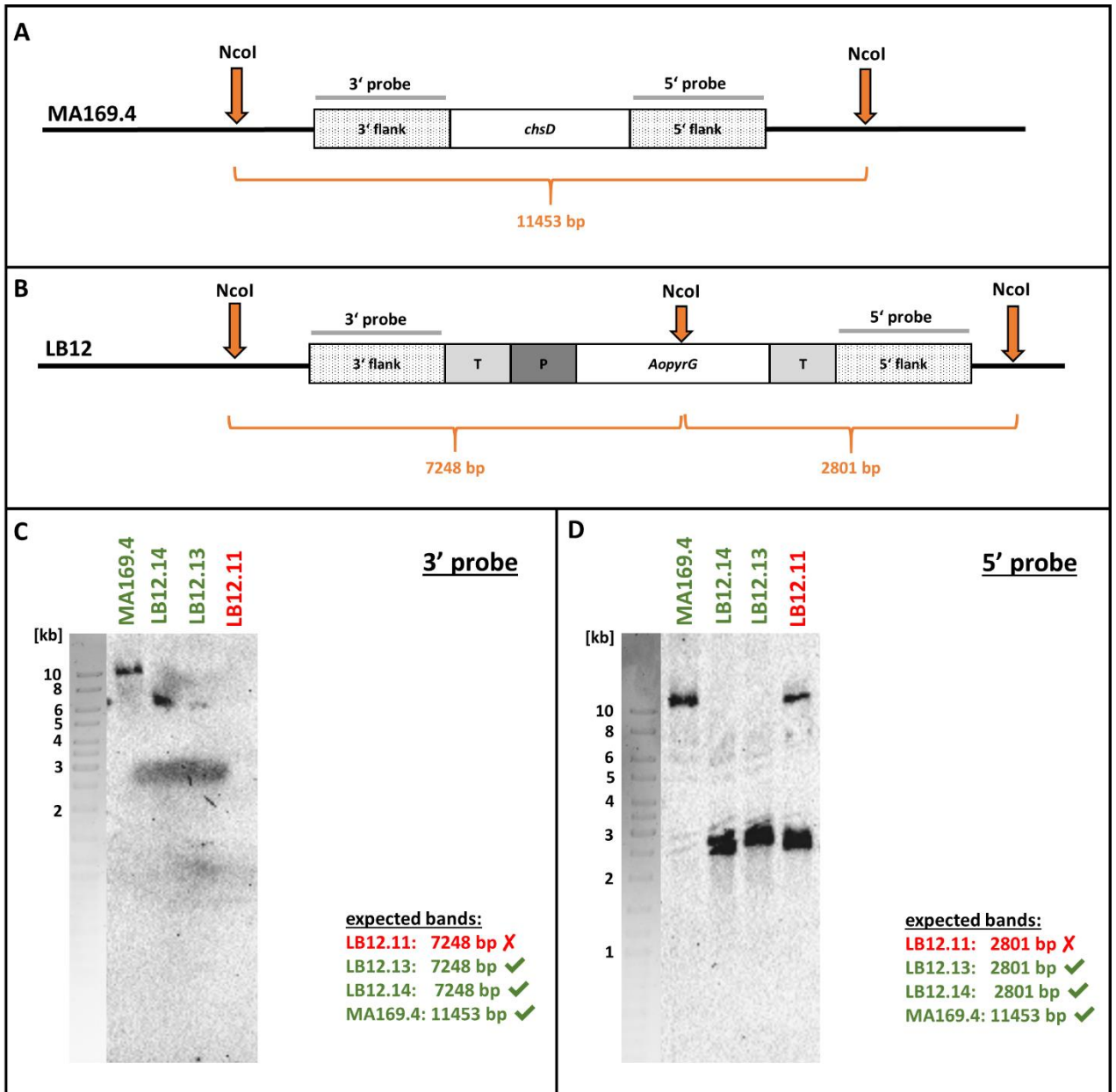

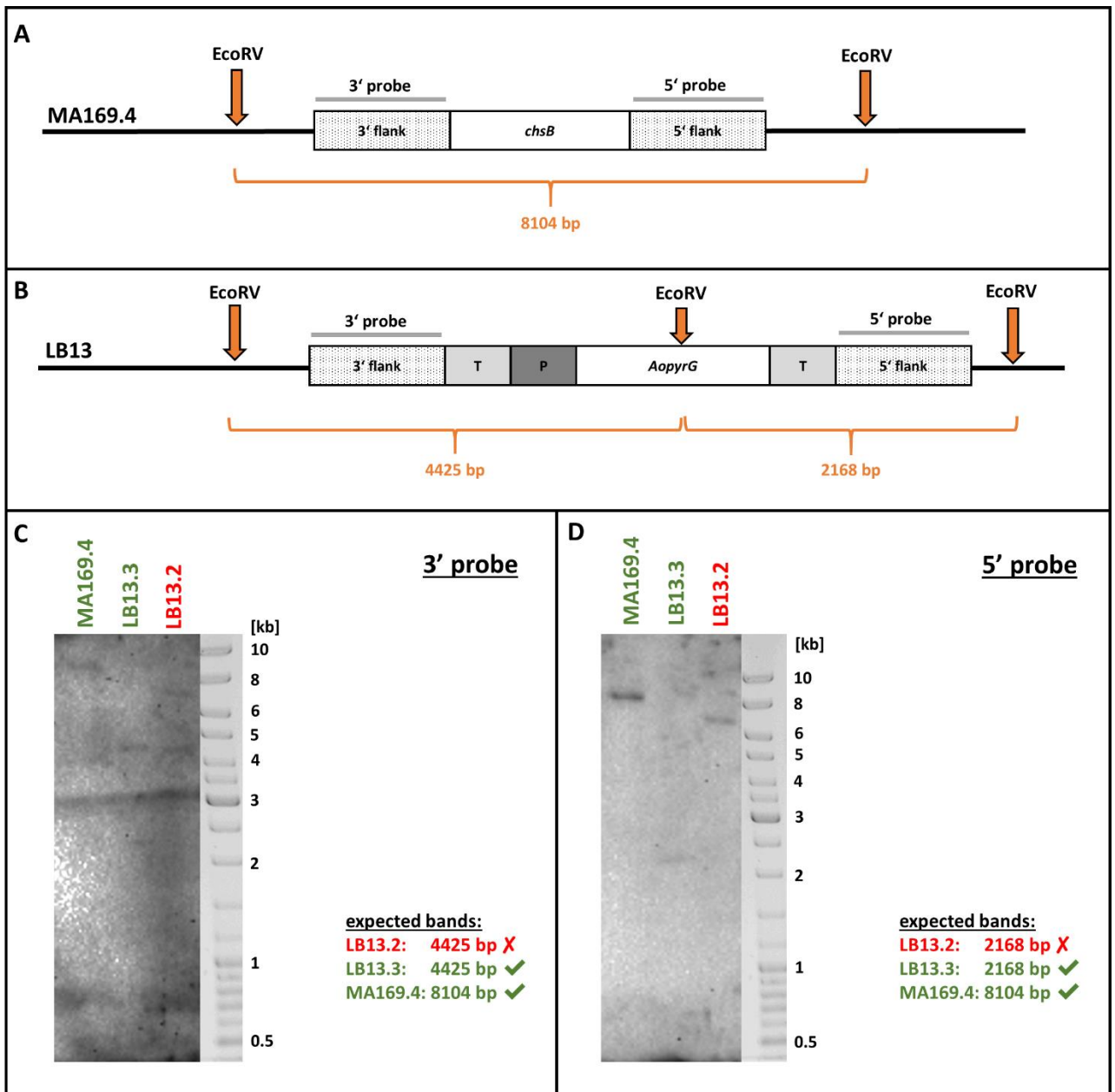

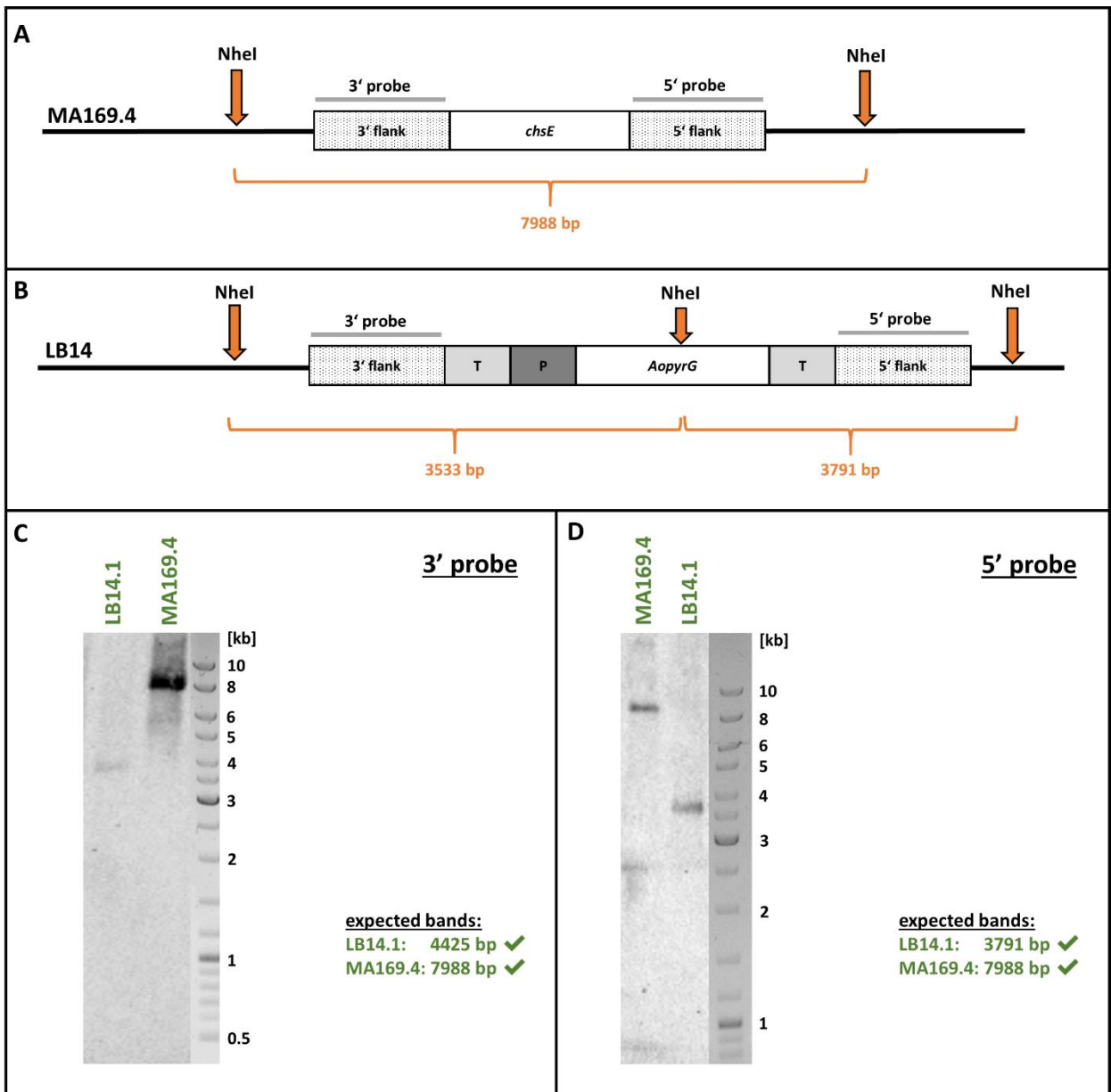

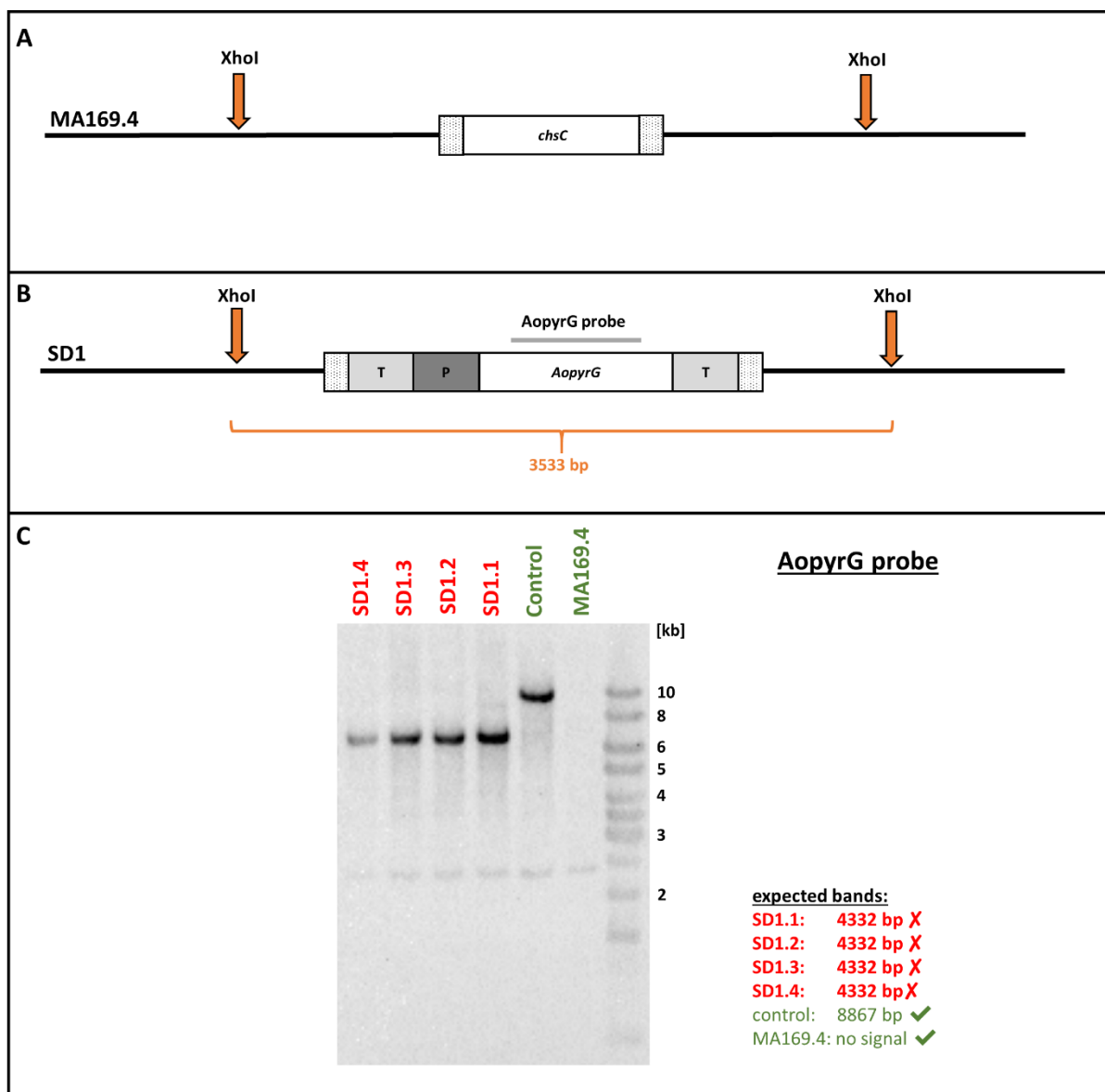

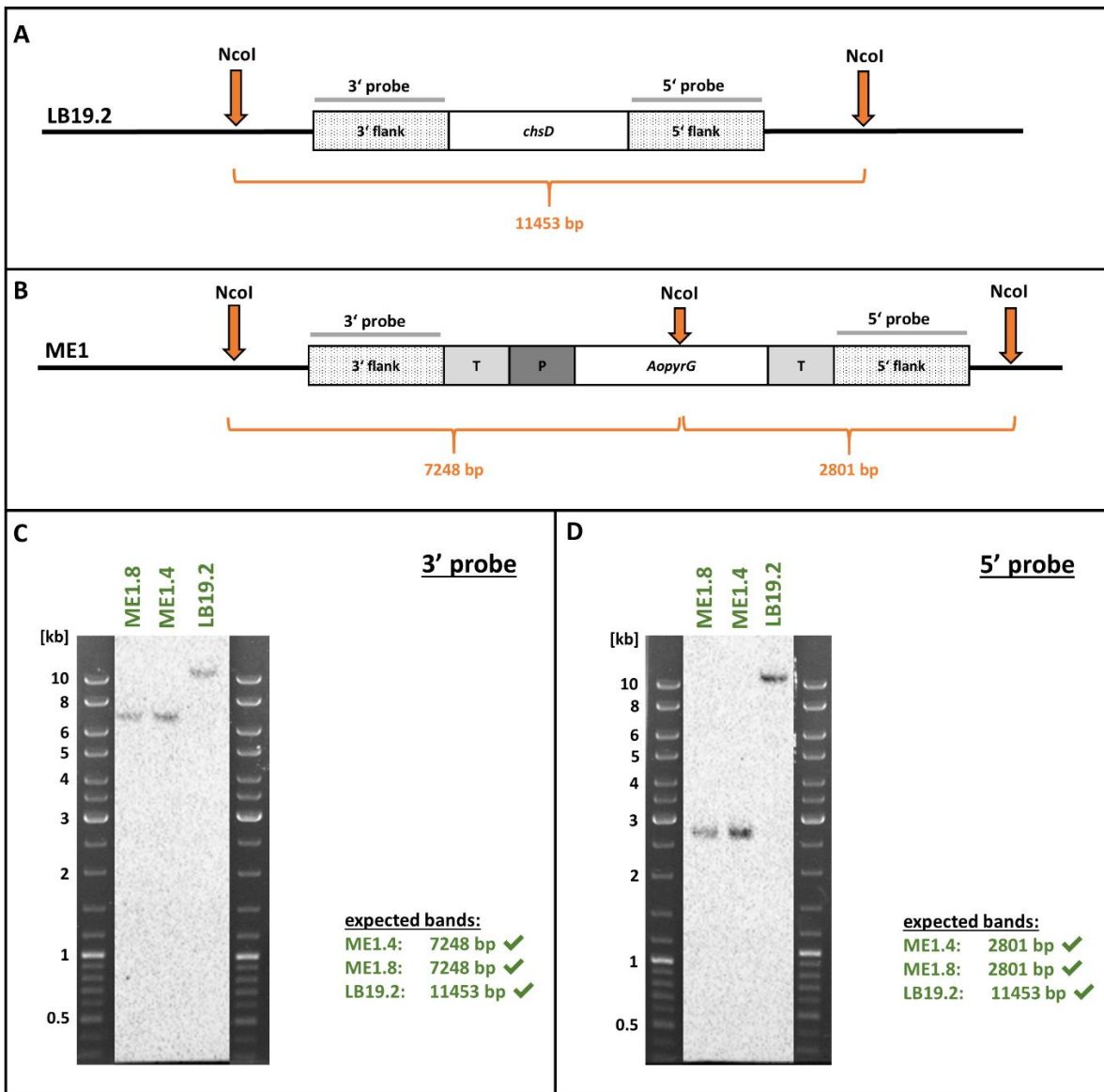

Supplement: Supplementary file 1 — Additional file 1: Figure S1. Schematic representation of protein domains in nine chitin synthase encoding genes predicted in A. niger. Figure S2. Expression of chitin synthase encoding genes across 155 conditions. Data were extracted from previous microarray analysis [18]. Figure S3. Word cloud depicting enriched GO terms (Biological Process) from chitin synthase encoding gene co-expression networks. Data were extracted from previous co-expression analysis [18]. Figure S4. Word cloud depicting enriched GO terms (Cellular Component) from chitin synthase encoding gene co-expression networks. Data were extracted from previous co-expression analysis [18]. Figure S5. Spearman correlation coefficients between chitin synthase encoding genes. Correlations >0.5 are shown. Data were extracted from previous co-expression analysis [18]. Figure S6. Southern blot confirmation of single cassette integration for A. niger transformants passing PCR quality control. Figure S7. Normalised radial colony growth rates of A. niger mutants on solid agar. 1000 spores were spotted onto MM agar +/- the indicated stress condition and incubated for 6 days at either 30 ℃ or 42 ℃. Colony diameters were measured and normalized to the MJK17.25 control at the respective condition. Table S1. Chitin synthase encoding genes predicted in A. niger CBS 513.88 assigned to various enzyme classes. The abbreviation recCHS describes a recombined chitin synthase. Table S2. Orthologues for A. niger chitin synthase encoding genes amongst indicated Aspergilli. ORF code, gene name and amino acid sequence homology compared to A. niger CBS 513.88 are listed. [file 40694_2024_172_MOESM1_ESM.zip › 40694_2024_172_MOESM1_ESM/Supplementary/Supplemental Figure S6.pdf]

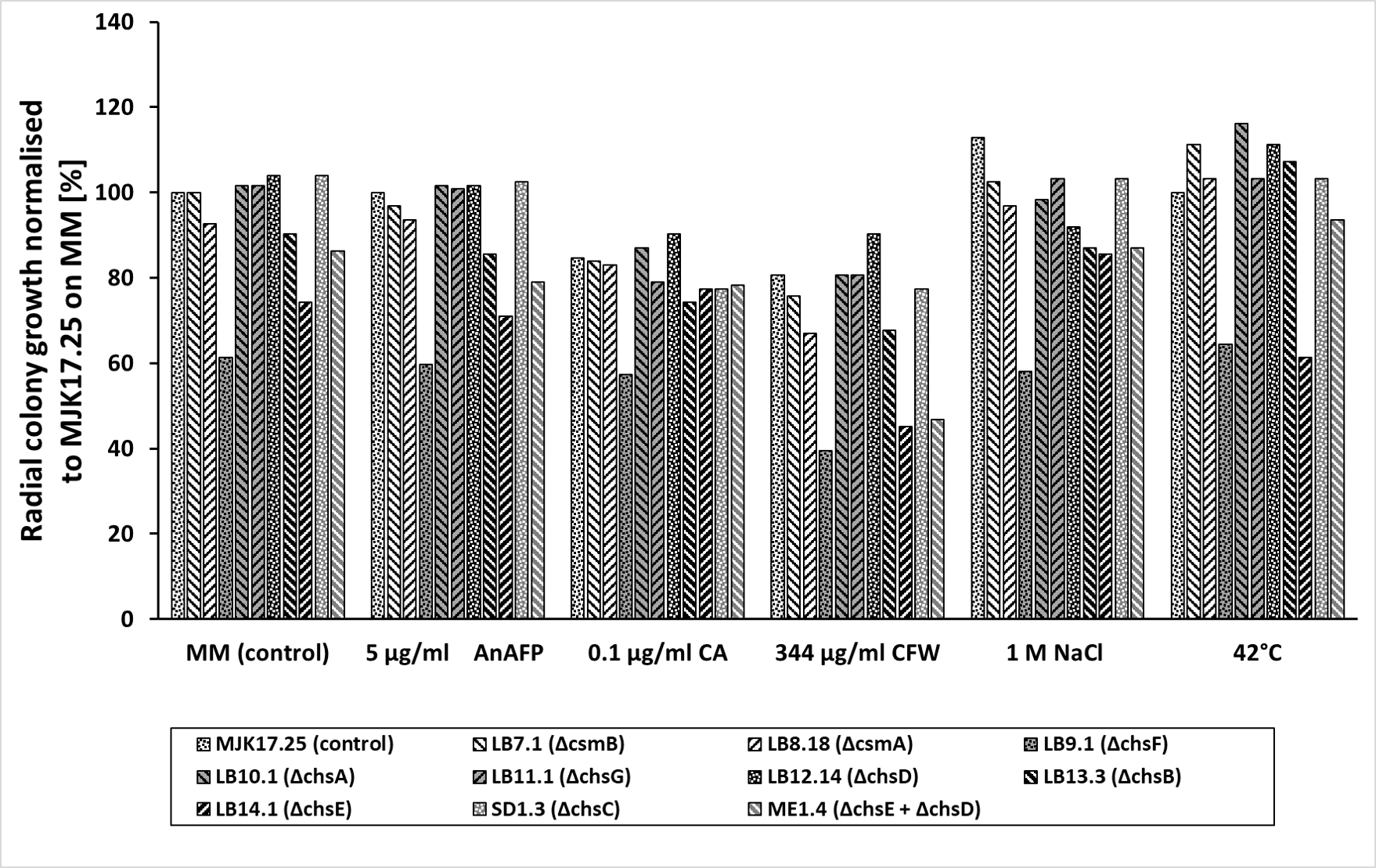

Supplement: Supplementary file 1 — Additional file 1: Figure S1. Schematic representation of protein domains in nine chitin synthase encoding genes predicted in A. niger. Figure S2. Expression of chitin synthase encoding genes across 155 conditions. Data were extracted from previous microarray analysis [18]. Figure S3. Word cloud depicting enriched GO terms (Biological Process) from chitin synthase encoding gene co-expression networks. Data were extracted from previous co-expression analysis [18]. Figure S4. Word cloud depicting enriched GO terms (Cellular Component) from chitin synthase encoding gene co-expression networks. Data were extracted from previous co-expression analysis [18]. Figure S5. Spearman correlation coefficients between chitin synthase encoding genes. Correlations >0.5 are shown. Data were extracted from previous co-expression analysis [18]. Figure S6. Southern blot confirmation of single cassette integration for A. niger transformants passing PCR quality control. Figure S7. Normalised radial colony growth rates of A. niger mutants on solid agar. 1000 spores were spotted onto MM agar +/- the indicated stress condition and incubated for 6 days at either 30 ℃ or 42 ℃. Colony diameters were measured and normalized to the MJK17.25 control at the respective condition. Table S1. Chitin synthase encoding genes predicted in A. niger CBS 513.88 assigned to various enzyme classes. The abbreviation recCHS describes a recombined chitin synthase. Table S2. Orthologues for A. niger chitin synthase encoding genes amongst indicated Aspergilli. ORF code, gene name and amino acid sequence homology compared to A. niger CBS 513.88 are listed. [file 40694_2024_172_MOESM1_ESM.zip › 40694_2024_172_MOESM1_ESM/Supplementary/Supplemental Figure S7.tif]

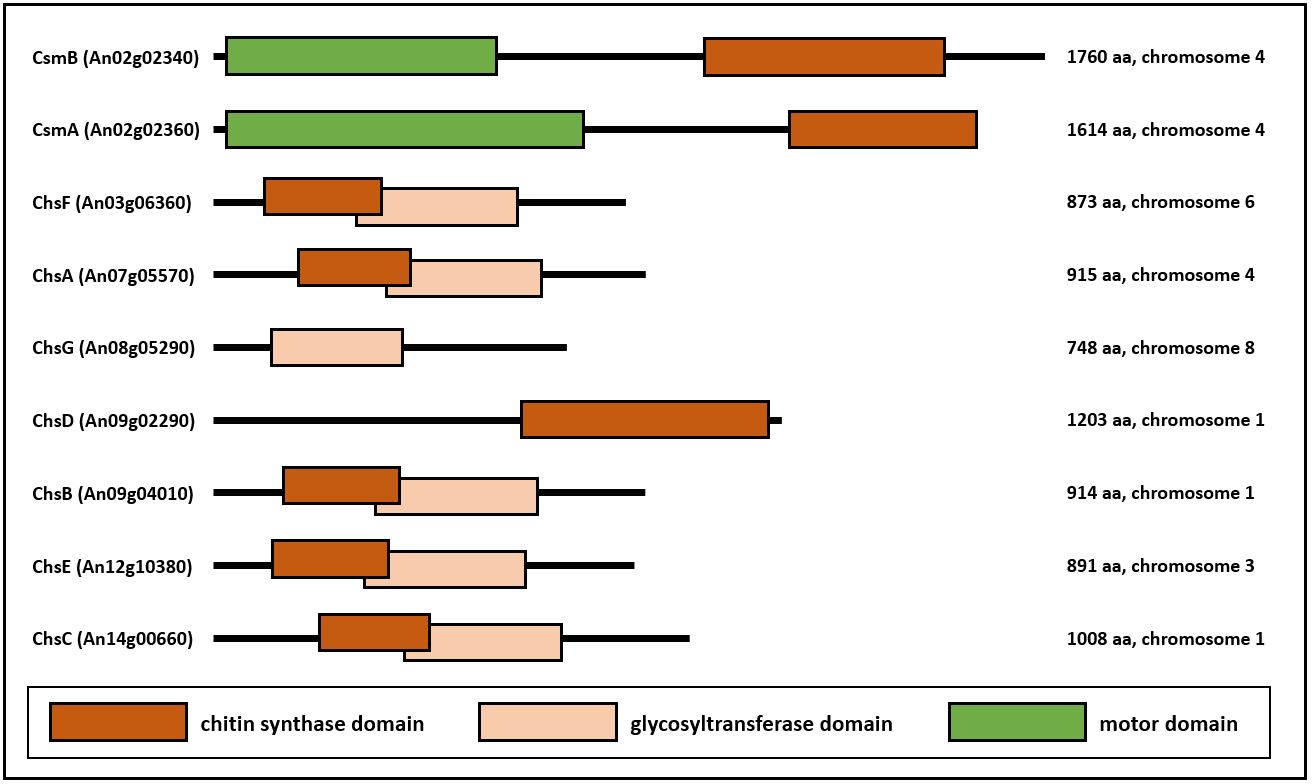

Supplement: Supplementary file 1 — Additional file 1: Figure S1. Schematic representation of protein domains in nine chitin synthase encoding genes predicted in A. niger. Figure S2. Expression of chitin synthase encoding genes across 155 conditions. Data were extracted from previous microarray analysis [18]. Figure S3. Word cloud depicting enriched GO terms (Biological Process) from chitin synthase encoding gene co-expression networks. Data were extracted from previous co-expression analysis [18]. Figure S4. Word cloud depicting enriched GO terms (Cellular Component) from chitin synthase encoding gene co-expression networks. Data were extracted from previous co-expression analysis [18]. Figure S5. Spearman correlation coefficients between chitin synthase encoding genes. Correlations >0.5 are shown. Data were extracted from previous co-expression analysis [18]. Figure S6. Southern blot confirmation of single cassette integration for A. niger transformants passing PCR quality control. Figure S7. Normalised radial colony growth rates of A. niger mutants on solid agar. 1000 spores were spotted onto MM agar +/- the indicated stress condition and incubated for 6 days at either 30 ℃ or 42 ℃. Colony diameters were measured and normalized to the MJK17.25 control at the respective condition. Table S1. Chitin synthase encoding genes predicted in A. niger CBS 513.88 assigned to various enzyme classes. The abbreviation recCHS describes a recombined chitin synthase. Table S2. Orthologues for A. niger chitin synthase encoding genes amongst indicated Aspergilli. ORF code, gene name and amino acid sequence homology compared to A. niger CBS 513.88 are listed. [file 40694_2024_172_MOESM1_ESM.zip › 40694_2024_172_MOESM1_ESM/Supplementary/Supplmenetal Figure S1.JPG]

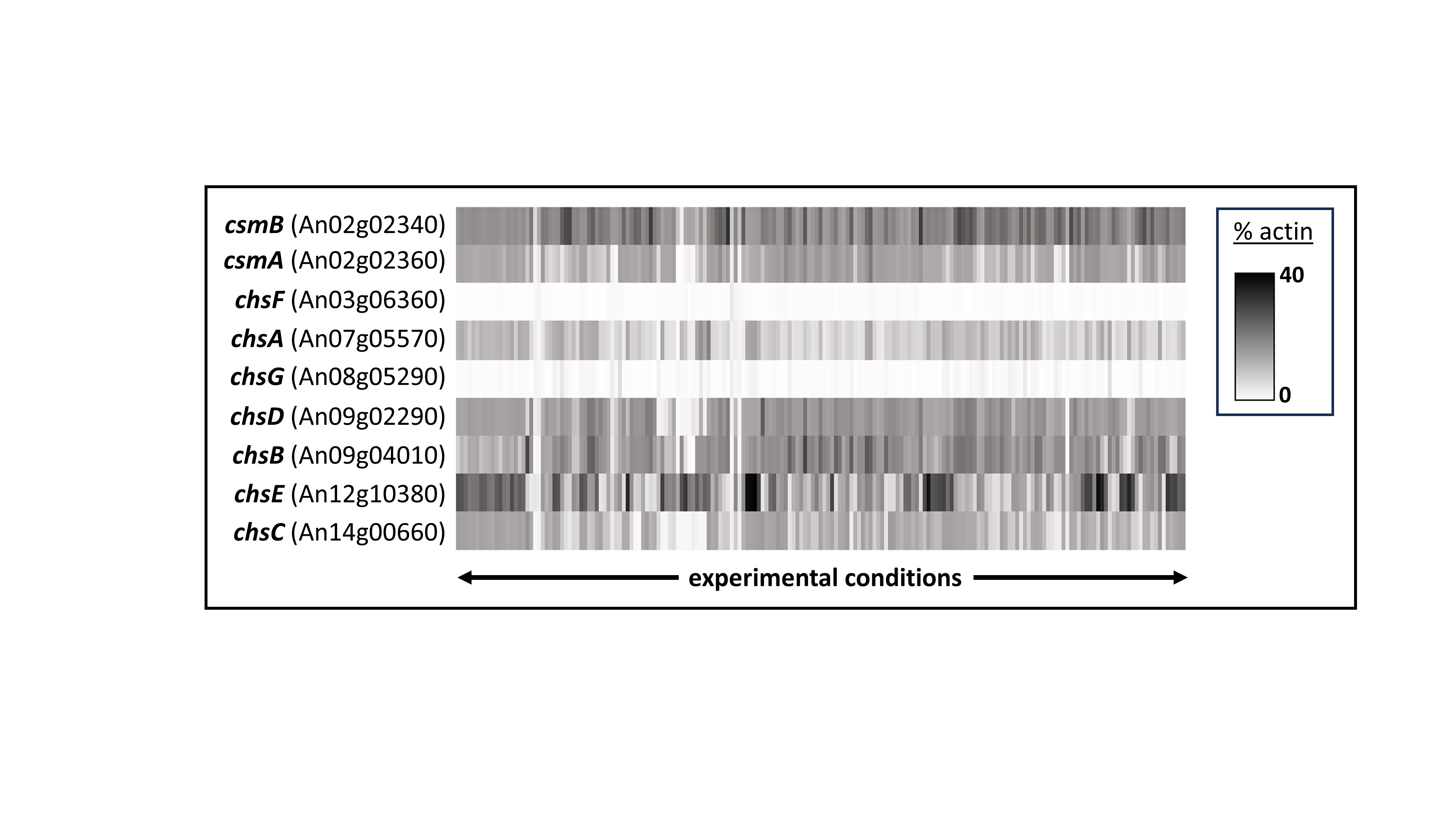

Supplement: Supplementary file 1 — Additional file 1: Figure S1. Schematic representation of protein domains in nine chitin synthase encoding genes predicted in A. niger. Figure S2. Expression of chitin synthase encoding genes across 155 conditions. Data were extracted from previous microarray analysis [18]. Figure S3. Word cloud depicting enriched GO terms (Biological Process) from chitin synthase encoding gene co-expression networks. Data were extracted from previous co-expression analysis [18]. Figure S4. Word cloud depicting enriched GO terms (Cellular Component) from chitin synthase encoding gene co-expression networks. Data were extracted from previous co-expression analysis [18]. Figure S5. Spearman correlation coefficients between chitin synthase encoding genes. Correlations >0.5 are shown. Data were extracted from previous co-expression analysis [18]. Figure S6. Southern blot confirmation of single cassette integration for A. niger transformants passing PCR quality control. Figure S7. Normalised radial colony growth rates of A. niger mutants on solid agar. 1000 spores were spotted onto MM agar +/- the indicated stress condition and incubated for 6 days at either 30 ℃ or 42 ℃. Colony diameters were measured and normalized to the MJK17.25 control at the respective condition. Table S1. Chitin synthase encoding genes predicted in A. niger CBS 513.88 assigned to various enzyme classes. The abbreviation recCHS describes a recombined chitin synthase. Table S2. Orthologues for A. niger chitin synthase encoding genes amongst indicated Aspergilli. ORF code, gene name and amino acid sequence homology compared to A. niger CBS 513.88 are listed. [file 40694_2024_172_MOESM1_ESM.zip › 40694_2024_172_MOESM1_ESM/Supplementary/Supplmenetal Figure S2.JPG]

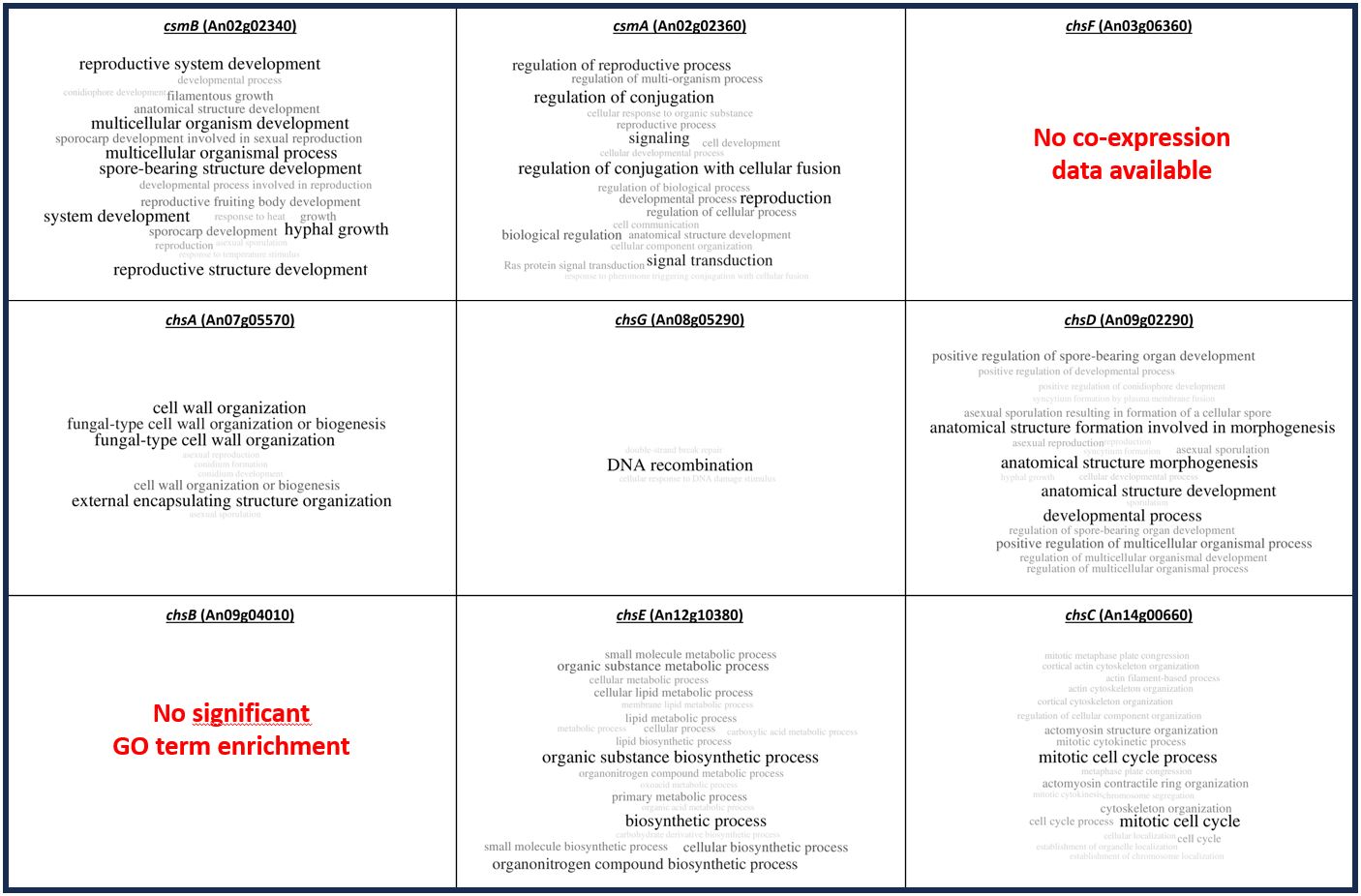

Supplement: Supplementary file 1 — Additional file 1: Figure S1. Schematic representation of protein domains in nine chitin synthase encoding genes predicted in A. niger. Figure S2. Expression of chitin synthase encoding genes across 155 conditions. Data were extracted from previous microarray analysis [18]. Figure S3. Word cloud depicting enriched GO terms (Biological Process) from chitin synthase encoding gene co-expression networks. Data were extracted from previous co-expression analysis [18]. Figure S4. Word cloud depicting enriched GO terms (Cellular Component) from chitin synthase encoding gene co-expression networks. Data were extracted from previous co-expression analysis [18]. Figure S5. Spearman correlation coefficients between chitin synthase encoding genes. Correlations >0.5 are shown. Data were extracted from previous co-expression analysis [18]. Figure S6. Southern blot confirmation of single cassette integration for A. niger transformants passing PCR quality control. Figure S7. Normalised radial colony growth rates of A. niger mutants on solid agar. 1000 spores were spotted onto MM agar +/- the indicated stress condition and incubated for 6 days at either 30 ℃ or 42 ℃. Colony diameters were measured and normalized to the MJK17.25 control at the respective condition. Table S1. Chitin synthase encoding genes predicted in A. niger CBS 513.88 assigned to various enzyme classes. The abbreviation recCHS describes a recombined chitin synthase. Table S2. Orthologues for A. niger chitin synthase encoding genes amongst indicated Aspergilli. ORF code, gene name and amino acid sequence homology compared to A. niger CBS 513.88 are listed. [file 40694_2024_172_MOESM1_ESM.zip › 40694_2024_172_MOESM1_ESM/Supplementary/Supplmenetal Figure S3.JPG]

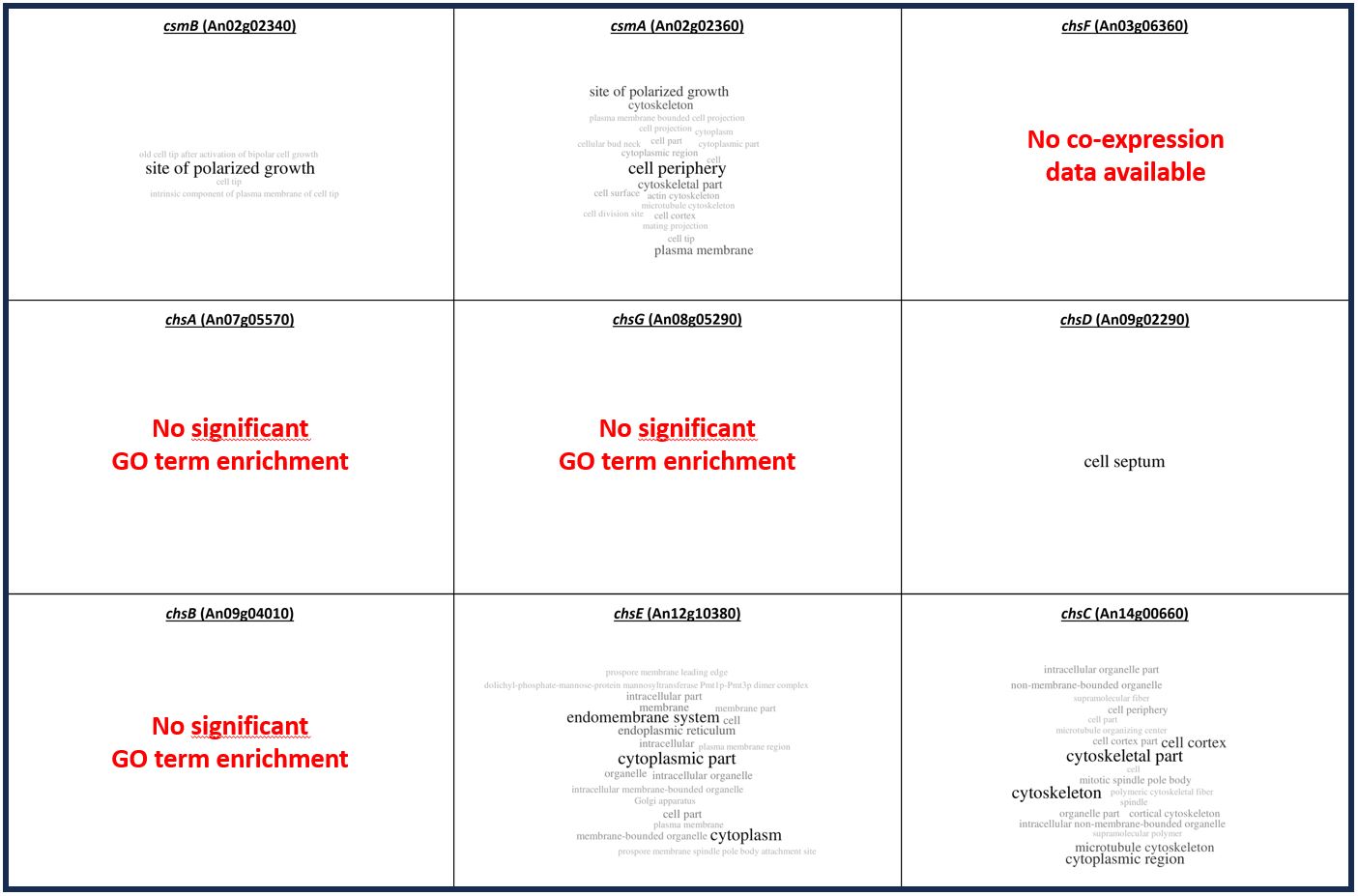

Supplement: Supplementary file 1 — Additional file 1: Figure S1. Schematic representation of protein domains in nine chitin synthase encoding genes predicted in A. niger. Figure S2. Expression of chitin synthase encoding genes across 155 conditions. Data were extracted from previous microarray analysis [18]. Figure S3. Word cloud depicting enriched GO terms (Biological Process) from chitin synthase encoding gene co-expression networks. Data were extracted from previous co-expression analysis [18]. Figure S4. Word cloud depicting enriched GO terms (Cellular Component) from chitin synthase encoding gene co-expression networks. Data were extracted from previous co-expression analysis [18]. Figure S5. Spearman correlation coefficients between chitin synthase encoding genes. Correlations >0.5 are shown. Data were extracted from previous co-expression analysis [18]. Figure S6. Southern blot confirmation of single cassette integration for A. niger transformants passing PCR quality control. Figure S7. Normalised radial colony growth rates of A. niger mutants on solid agar. 1000 spores were spotted onto MM agar +/- the indicated stress condition and incubated for 6 days at either 30 ℃ or 42 ℃. Colony diameters were measured and normalized to the MJK17.25 control at the respective condition. Table S1. Chitin synthase encoding genes predicted in A. niger CBS 513.88 assigned to various enzyme classes. The abbreviation recCHS describes a recombined chitin synthase. Table S2. Orthologues for A. niger chitin synthase encoding genes amongst indicated Aspergilli. ORF code, gene name and amino acid sequence homology compared to A. niger CBS 513.88 are listed. [file 40694_2024_172_MOESM1_ESM.zip › 40694_2024_172_MOESM1_ESM/Supplementary/Supplmenetal Figure S4.JPG]

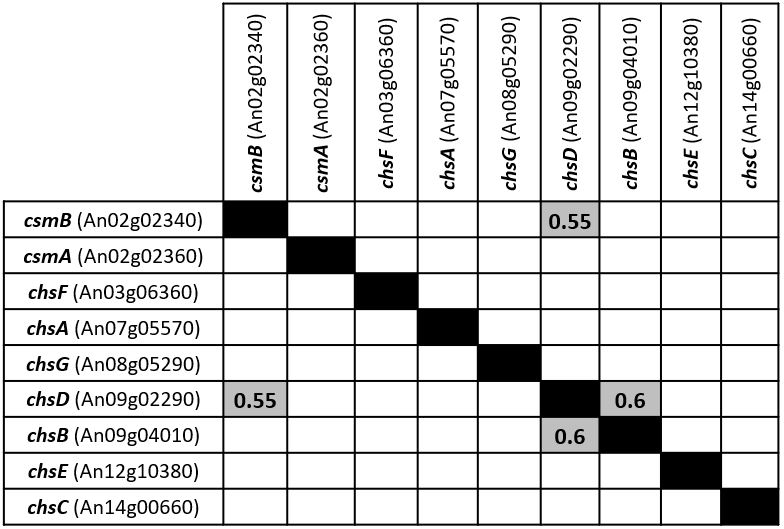

Supplement: Supplementary file 1 — Additional file 1: Figure S1. Schematic representation of protein domains in nine chitin synthase encoding genes predicted in A. niger. Figure S2. Expression of chitin synthase encoding genes across 155 conditions. Data were extracted from previous microarray analysis [18]. Figure S3. Word cloud depicting enriched GO terms (Biological Process) from chitin synthase encoding gene co-expression networks. Data were extracted from previous co-expression analysis [18]. Figure S4. Word cloud depicting enriched GO terms (Cellular Component) from chitin synthase encoding gene co-expression networks. Data were extracted from previous co-expression analysis [18]. Figure S5. Spearman correlation coefficients between chitin synthase encoding genes. Correlations >0.5 are shown. Data were extracted from previous co-expression analysis [18]. Figure S6. Southern blot confirmation of single cassette integration for A. niger transformants passing PCR quality control. Figure S7. Normalised radial colony growth rates of A. niger mutants on solid agar. 1000 spores were spotted onto MM agar +/- the indicated stress condition and incubated for 6 days at either 30 ℃ or 42 ℃. Colony diameters were measured and normalized to the MJK17.25 control at the respective condition. Table S1. Chitin synthase encoding genes predicted in A. niger CBS 513.88 assigned to various enzyme classes. The abbreviation recCHS describes a recombined chitin synthase. Table S2. Orthologues for A. niger chitin synthase encoding genes amongst indicated Aspergilli. ORF code, gene name and amino acid sequence homology compared to A. niger CBS 513.88 are listed. [file 40694_2024_172_MOESM1_ESM.zip › 40694_2024_172_MOESM1_ESM/Supplementary/Supplmenetal Figure S5.JPG]
